# Hyaluronic acid synthase 2 dysfunction exacerbates elastase-induced neutrophilic airway inflammation and emphysema in mice

**DOI:** 10.3389/fimmu.2025.1683385

**Published:** 2025-10-14

**Authors:** Kengo Nishino, Takumi Kiwamoto, Masashi Matsuyama, Zhenting Wei, Sosuke Matsumura, Kenya Kuramoto, Yuki Yabuuchi, Kai Yazaki, Kazufumi Yoshida, Yosuke Matsuno, Yuko Morishima, Nobuyuki Hizawa

**Affiliations:** ^1^ Department of Pulmonary Medicine, Institute of Medicine, University of Tsukuba, Tsukuba, Japan; ^2^ Department of Pulmonary Medicine, Nikko Memorial Hospital, Hitachi, Japan

**Keywords:** HAS2, chronic obstructive pulmonary disease, emphysema, TGF-β1, G-CSF, neutrophil

## Abstract

Chronic obstructive pulmonary disease (COPD) is a progressive inflammatory lung disorder primarily caused by prolonged exposure to harmful substances, such as cigarette smoke. Hyaluronic acid synthase 2 (HAS2) synthesizes high-molecular-weight hyaluronic acid (HMW-HA), which has anti-inflammatory properties. Previous studies have revealed that HAS2 dysfunction may exacerbate COPD. However, the specific impact of HAS2 on pulmonary emphysema progression remains unclear. Therefore, this study examined whether HAS2 dysfunction worsens airway inflammation and emphysema in a mouse COPD model. *Has2* heterozygous-deficient (*Has2* ^+/−^) mice and their wild-type (WT) littermates were evaluated using a porcine pancreatic elastase (PPE)-induced COPD model. After the administration of PPE, the *Has2* ^+/−^ mice exhibited a significant increase in total cell and neutrophil counts in the bronchoalveolar lavage fluid samples compared with the WT mice. Further, the *Has2* ^+/−^ mice presented with enhanced emphysema development on histological analyses, with higher mean linear intercept values relative to the WT mice. The PPE-stimulated *Has2* ^+/−^ mice also had increased G-CSF levels and tumor growth factor-beta (TGF-β) attenuation in the lungs. RNA-sequencing analysis revealed that PPE stimulation promoted the synthesis of HMW-HA and TGF-β signaling. Gene Ontology analysis using *Has2* ^+/−^ mice-specific differentially expressed genes showed that the genes associated with the pathways that promote the negative regulation of the TGF-β receptor signaling pathway were activated after the administration of PPE. Therefore, *Has2* dysfunction exacerbates neutrophilic airway inflammation and emphysema, thereby underscoring the protective role of HAS2 in a PPE-induced emphysema model. The exacerbated response may involve G-CSF- and TGF-β-related signaling pathways. These findings may contribute to the development of novel therapeutic strategies for COPD management.

## Introduction

Chronic obstructive pulmonary disease (COPD) is a heterogeneous lung condition characterized by chronic respiratory symptoms attributed to abnormalities of the airways and/or alveoli that cause persistent airflow obstruction (1). Further, it is one of the leading causes of mortality worldwide (2–4). The financial implications of COPD management are projected to increase further on a global scale (5). Therefore, the early identification of patients at risk for rapid COPD progression is important in reducing the disease-related costs. COPD is characterized anatomically by small airway remodeling and emphysema. In pulmonary emphysema, elastin is the major target of decomposition leading to loss of lung elasticity (6, 7). A previous study has shown that hyaluronan (HA) binds to elastic fibers and protects them from elastase-induced injury (8, 9). Another study found that patients with asthma or COPD had lower levels of HA in their airway smooth muscle cells (10). This study also revealed a significant decrease in the HA synthase 1 (HAS1) and HAS2 levels in the same tissue (10). HA synthases synthesize HA polymers of various sizes. HAS1 and HAS2 produce high-molecular-weight HA (HMW-HA, 2 × 10^4^ kDa). Meanwhile, HAS3 synthesizes low-molecular-weight HA (2 × 10^2^ kDa) (11). Our previous genome-wide association study has found that the *HAS2* gene is a potential susceptibility factor for adult asthma (12). Using HAS2 gene (*Has2*) heterozygous-deficient (*Has2* ^+/−^) mice, we also found that the attenuation of *Has2* exacerbates eosinophilic airway inflammation, increases airway hyperresponsiveness, and promotes airway remodeling (13, 14). HMW-HA has anti-inflammatory effects. However, it remains unclear whether the dysfunction of *Has2*, which produces HMW-HA, contributes to the severity and intractability of other inflammatory lung diseases such as COPD. To validate the role of *Has2* in the pathogenesis of COPD, this study analyzed the development of porcine pancreatic elastase-induced pulmonary emphysema in *Has2* ^+/−^ mice. Results showed that the *Has2* ^+/−^ mice exhibited a more severe neutrophilic airway inflammatory reaction, higher degree of emphysema formation, and increased granulocyte-colony stimulating factor (G-CSF) with transforming growth factor beta 1 (TGF-β1) attenuation.

## Methods

### Animals

The experiments used 8–12-week-old female C57BL/6 background wild-type (WT) mice and *Has2* heterozygous (*Has2* ^+/-^) mice. Breeding sets of *Has2* ^+/−^ mice (Jackson Laboratory, Bar Harbor, ME) were backcrossed to the C57BL/6 background for at least 10 generations (13–15). The Institutional Review Board of the University of Tsukuba approved all animal studies (approval numbers: 21-028, 23-004, and 24-057).

### Elastase inoculation

Mice were injected intraperitoneally with triple mixed anesthesia of medetomidine hydrochloride (0.30 mg/kg; Nippon Zenyaku Kogyo, Fukushima, Japan), midazolam (4 mg/kg; Fujifilm Wako Pure Chemical, Osaka, Japan), and butorphanol tartrate (5 mg/kg; Meiji Animal Health, Kumamoto, Japan). After induction of anesthesia, the mice were inoculated with 3.75 U of porcine pancreatic elastase (PPE; Elastin Products Company, Owensville, MO) in 100 μl of saline intratracheally. After the administering PPE, atipamezole hydrochloride (0.30 mg/kg; Nippon Zenyaku Kogyo) was injected intraperitoneally to awaken the mice. The mice were sacrificed by exsanguination through the abdominal aorta under anesthesia on days 1, 4, and 21. An age-matched PPE non-treated mice group was used as the control group.

### Experimental groups

Animals were randomly divided into four groups:

WT-Control: WT mice maintained under filtered air without the administration of PPE (n = 8).

*Has2* ^+/−^-Control: *Has2* ^+/−^ mice maintained under filtered air without the administration of PPE (n = 8).

WT-PPE: WT mice maintained under filtered air that received PPE administration (n = 8 for each time point).

*Has2* ^+/−^-PPE: *Has2* ^+/−^ mice maintained under filtered air that received PPE administration (n = 8 for each time point).

To avoid the influence of each measurement value during specimen collection, three experimental setups were necessary for the protocol. In the first, lungs (right and left) were used to conduct morphometric analysis. The second setup was required to perform bronchoalveolar lavage fluid cell analysis. The left lungs from the third set up were required to obtain lung homogenates for cytokine analysis. RNA-seq analysis was performed on the right lung samples from the third setup.

### Lung histology

Lung paraffin sections were stained with hematoxylin and eosin (HE) staining to assess air space enlargement and airway inflammatory cell infiltration. The mean linear intercept (MLI) was quantified, as described in a previous study (16). Briefly, we prepared at least four slides from both the side lung and performed MLI analysis in the distal regions of the parenchymal tissue. For each prepared section, ten fields of view (500 μm × 373.5 μm each) of peripheral airspaces that were free of blood vessels and bronchioles were selected. The MLI was then calculated based on the average number of intersections between ten horizontally spaced lines, which were placed equidistant from each other and the alveolar walls within all field of view.

### Bronchoalveolar lavage fluid cell counting

Mouse lungs were lavaged using five repeated instillations of 0.6 mL of saline each via the tracheal cannula. The recovery rate was between 75 and 85%. The cell count was determined using a hemocytometer, and a differential cell count was subsequently performed based on count of 300 cells. The cells were morphologically classified using staining with Diff-Quik (Polysciences, Inc., Warrington, PA), using standard light microscopic techniques.

### Enzyme-linked immunosorbent assay

Quantification of TGF-β1 in the lung homogenates was performed using commercially available ELISA kits (R&D Systems, Minneapolis, MN).

### Multiplex cytokine assay

Cytokines and chemokines in the lung homogenates were measured using the MILLIPLEX MAP Kit (MilliporeSigma, Burlington, MA), according to the manufacturer’s instructions.

### Lung RNA extraction and RNA sequencing

Total RNA was extracted from the mouse lungs using the TRIzol^®^ reagent (Thermo Fisher Scientific, Waltham, MA), according to the manufacturer’s instructions (n = 6–7, each group). The concentration and purity of the RNA samples were determined via automated optical density evaluation (OD 260/OD 280 ≥ 1.8 and OD 260/OD 230 ≥ 1.8) using the NanoDrop spectrophotometer (Thermo Fisher Scientific). RNA sequencing (RNA-seq) libraries were prepared using the NEBNext rRNA Depletion Kit (New England Biolabs, Ipswich, MA) and the ENBNext Ultra Directional RNA Library Prep Kit (New England Biolabs), according to the manufacturer’s instructions, using 500 ng of total RNA samples. Next, 2 × 36 base paired-end sequencing was performed using the NextSeq 500 sequencer (Illumina, San Diego, CA) by Tsukuba i-Laboratory LLP (Tsukuba, Japan). Sequences were mapped to the mm10 mouse genome and quantified using CLC Genomics Workbench version 10.1.1 (QIAGEN, Hilden, Germany). Differentially expressed genes were identified by filtering according to the P-values obtained via analysis of variance (Gaussian Statistical Analysis in CLC). The data are available under GEO series accession number GSE 304417.

### Pathway analysis of DEGs and CIBERSORT analysis

The biosynthesis and catabolism of the hyaluronan glycosylation pathway were visualized using GlycoMaple (https://glycosmos.org/glycomaple/Human) (**Supplementary Figure 1**). Upstream analysis was performed on RNA-seq data using the Ingenuity Pathway Analysis software (QIAGEN) with the Fisher’s exact test (P < 0.05, indicates statistically significant differences). Unique differentially expressed genes (DEGs) between WT-Control vs WT-PPE and WT-Control vs *Has2* ^+/–^PPE were identified using Venny (v2.0; http://bioinfogp.cnb.csic.es/tools/venny/index.html). The Gene Ontology (GO) terms enriched in the DE genes were identified using Metascape (http://metascape.org). CIBERSORT analysis was performed on the RNA-seq data using the analytical tool (https://cibersort.stanford.edu/) (17). A previously published mouse reference signature matrix, comprising 511 distinguishing genes for 25 immune cell types, was used as a reference profile (18).

### Statistical analysis

Data were presented as means ± standard error of the means or individual dot plots with means ± standard error of the means. Between-group differences were evaluated using the Mann–Whitney U test or analysis of variance with the Tukey’s multiple comparison test. P values < 0.05 indicated statistically significant differences.

## Results

### The development of elastase-induced pulmonary emphysema was enhanced in *Has2^+/−^* mice

**Figure 1:**
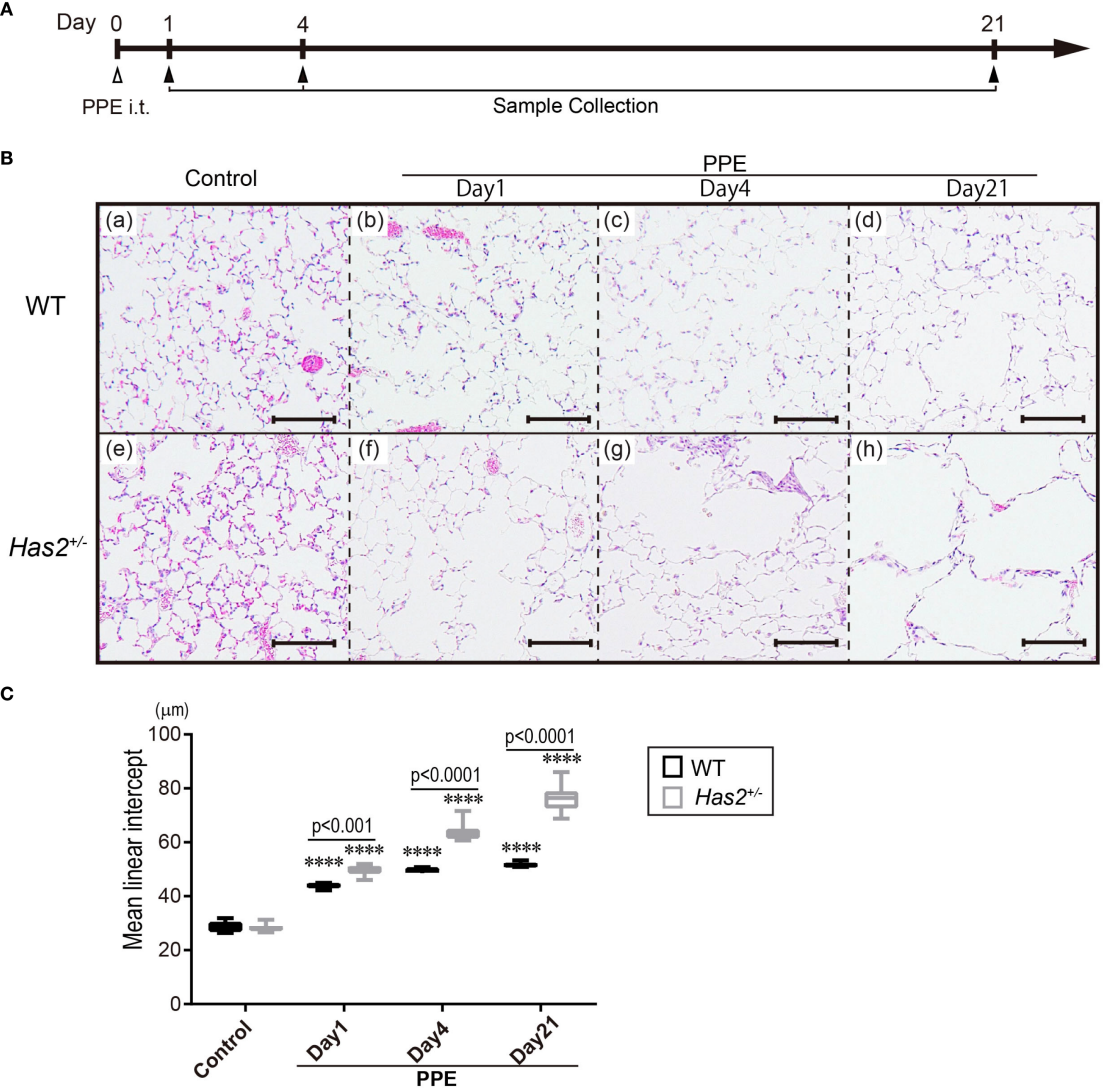
*Has2* attenuation exacerbated lung emphysema in a PPE-induced mouse model of COPD. **(A)** Schematic illustration of the experimental design. **(B)** Lung tissue HE staining in the WT mice (upper lane, panels a–d) and the *Has2* ^+/−^ mice (lower lane, panels e–h). Scale bar: 100 μm. **(C)** Mean linear intercept (MLI) values of the alveoli (n = 8). Statistical significance was determined using the Tukey’s multiple comparison test. ****P < 0.0001 relative to the WT-Control mice. Horizontal bars indicate direct statistical comparisons between WT and *Has2* ^+/−^ mice.

To elucidate the protective role of HAS2 against emphysema, we initially evaluated the development of emphysema 1, 4, and 21 days after the administration of PPE (**Figure 1A**). Pathological differences were not observed between WT and *Has2* ^+/−^ mice before PPE treatment upon microscopic examination (**Figure 1B**, panels a and e). Air space enlargement and alveolar wall disruption worsened over time after PPE treatment in both WT (**Figure 1B**, upper panel) and *Has2* ^+/−^ mice (**Figure 1B**, lower panel). However, these pathological changes were more severe in *Has2* ^+/−^ mice than in WT mice. To quantify morphologic changes, MLI was then evaluated. Morphometric analysis revealed that the *Has2* ^+/−^ mice had significantly higher MLI values than the WT mice on days 1, 4, and 21 after the administration of PPE (**Figure 1C**). Taken together, these histological evaluations have revealed that *Has2* ^+/−^ mice are more susceptible to PPE-induced emphysema.

### The severity and duration of elastase-induced pulmonary inflammation were enhanced in *Has2* ^+/−^ mice

**Figure 2:**
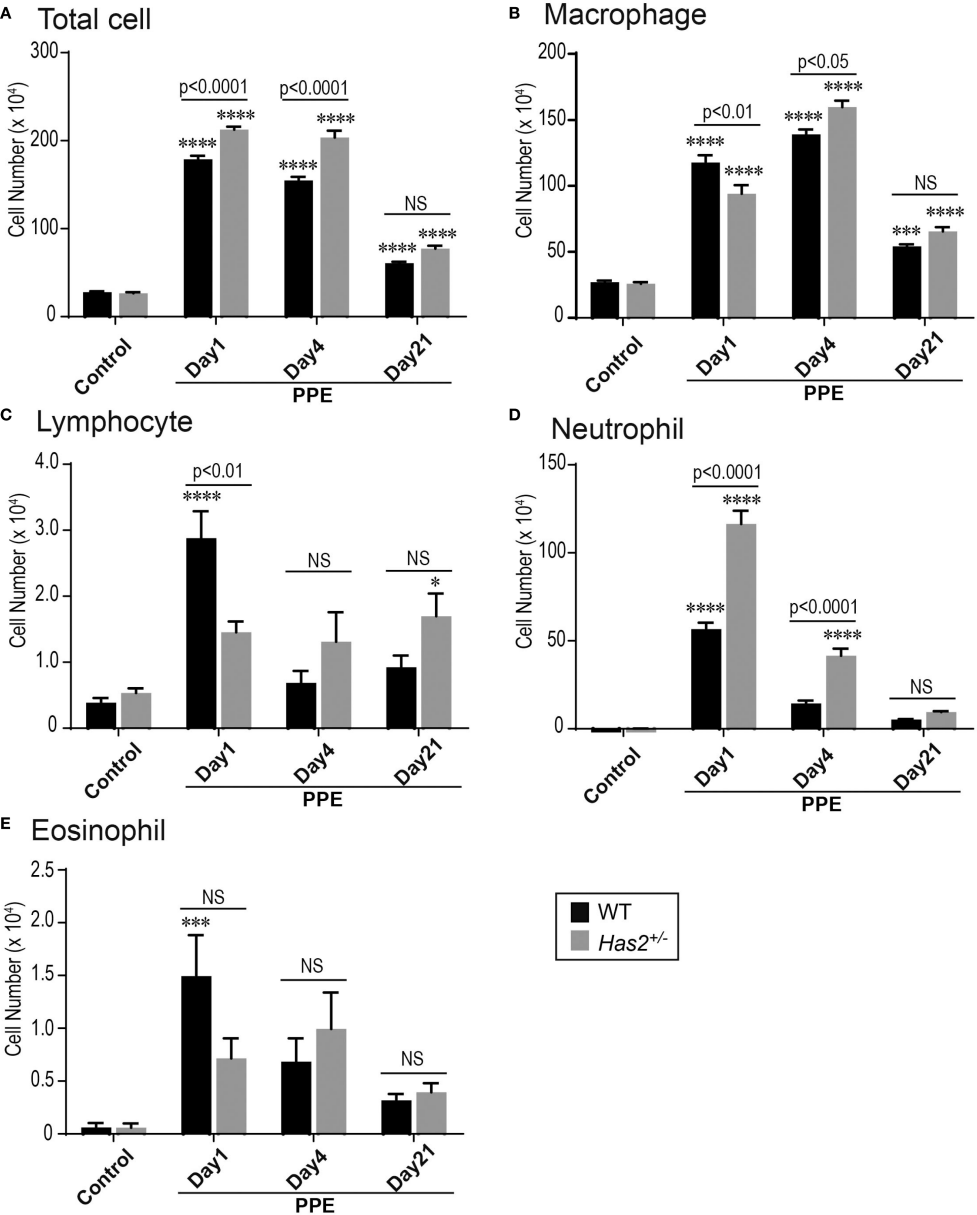
*Has2* attenuation exacerbated acute neutrophilic airway inflammation in a PPE-induced mouse model of COPD. BALF cytology of each indicated cell type (n = 8). **(A)** Total cell. **(B)** Macrophage. **(C)** Lymphocyte. **(D)** Neutrophil. **(E)** Eosinophil. Statistical significance was determined using the Tukey’s multiple comparison test. * P < 0.05, *** P < 0.001, **** P < 0.0001 relative to the WT-Control mice. Horizontal bars indicate direct statistical comparisons between WT and *Has2* ^+/−^ mice. NS, not significant.

To examine the severity of pulmonary inflammation induced by elastase, the number of inflammatory cells in the bronchoalveolar lavage fluid (BALF) was determined. Both the WT-PPE and *Has2* ^+/−^-PPE mice had a significantly higher number of total cells and macrophages than the WT-Control mice (**Figures 2A, B**). Leukocyte subsets were analyzed to characterize inflammation in WT-PPE and *Has2* ^+/−^-PPE mice. The number of BALF neutrophils on day 1 was significantly higher in the *Has2* ^+/−^ mice than in the WT mice (**Figure 2D**). In the *Has2* ^+/−^ mice, the significant increase in the neutrophil counts persisted 4 days after the administration of PPE (**Figure 2D**). The WT-PPE mice had a higher number of lymphocytes and eosinophils on day 1 than the *Has2* ^+/−^-PPE mice (**Figures 2C, E**). This phenomenon could be attributed to the fact that the absolute numbers of lymphocytes and eosinophils were considerably lower than the absolute number of neutrophils. These results indicate that *Has2* attenuation worsens PPE-induced neutrophilic airway inflammation.

### *Has2* attenuation results in the downregulation of TGF-β1

**Figure 3:**
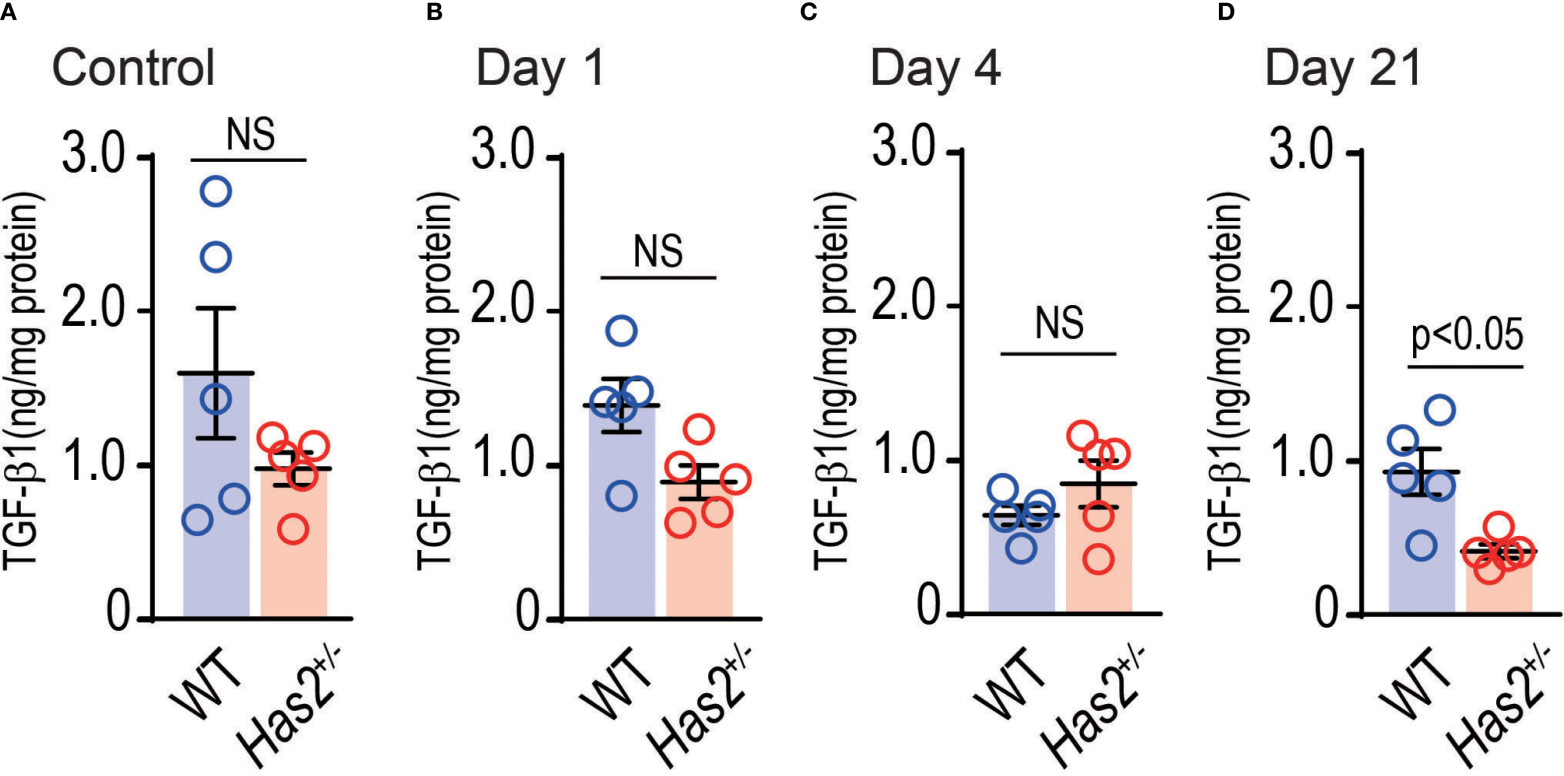
Effects of *Has2* attenuation on the TGF-β1 levels in the lung homogenates. Protein adjusted levels of TGF-β1 (n = 5). **(A)** Control. **(B)** Day 1. **(C)** Day4. **(D)** Day 21. Statistical significance was determined using the Mann–Whitney U test. Horizontal bars indicate direct statistical comparisons between WT and *Has2* ^+/−^− mice. NS, not significant.

TGF-β is known to be regulated by CD44, a HA-binding protein (19). To determine whether *Has2* attenuation affects the expression of lung TGF-β1, the TGF-β1 levels in the lung homogenate were evaluated. As shown in **Figure 3**, the protein-adjusted levels of TGF-β1 in the lung homogenates of *Has2* ^+/−^ mice were more likely to be lower than those of WT mice. These levels decreased significantly 21 days after the administration of PPE (**Figure 3D**), as determined using the Mann–Whitney U test. These results may support the hypothesis that *Has2* attenuation prolongs the inhibitory state of HA-binding protein expression and TGF-β signaling.

### Elastase-stimulated *Has2^+/−^* mice exhibit increased G-CSF levels in the lung

**Figure 4:**
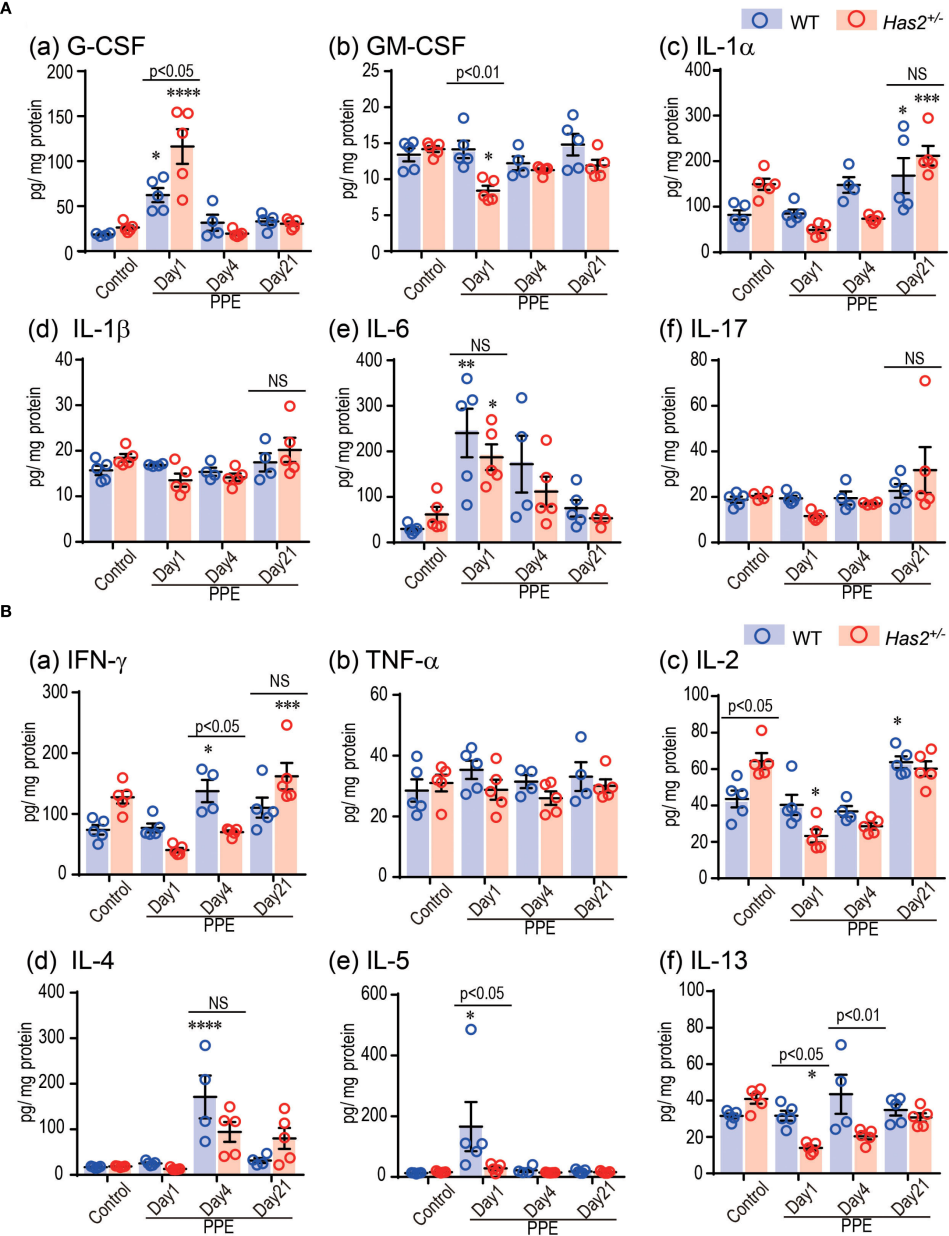
Effects of *Has2* attenuation on various cytokine and chemokine levels in the lung homogenates. **(A)** Protein adjusted levels of inflammatory cytokines in the lung homogenates (n = 4–5). **(B)** Protein adjusted levels of the indicated Th1-/Th2-related cytokines in the lung homogenates (n = 4–5). Statistical significance was determined using the Tukey’s multiple comparison test. *P < 0.05, **P < 0.01, ***P < 0.001, ****P < 0.0001 relative to the WT-Control mice. Horizontal bars indicate direct statistical comparisons between WT and *Has2* ^+/−^ mice. NS, not significant.

To examine the hypothesis that significant lung neutrophilia and emphysema progression in *Has2* ^+/−^ mice was attributed to an altered inflammatory cytokine and chemokine response, lung homogenate samples were tested for a variety of potentially relevant mediators (**Figure 4**, **Supplementary Figures 2, 3**). The *Has2* ^+/−^-PPE mice had significantly higher G-CSF levels in the lung homogenates than the WT-PPE mice on day 1 (**Figure 4A**, panel a). Conversely, the *Has2* ^+/−^-PPE mice had significantly lower levels of GM-CSF, IL-5, IL-7, IL-10, IL-12(p70), IL-13, and IL-15 than the WT-PPE mice on day 1 (**Figure 4**, **Supplementary Figures 2, 3**). The *Has2* ^+/−^-PPE group had significantly higher protein concentrations than the WT-PPE group on day 1. Therefore, this difference might have affected the results of the protein adjusted cytokine and chemokine measurements (**Supplementary Figures 4**). Interestingly, on day 4, the *Has2* ^+/−^-PPE group had significantly lower levels of IFN-γ, and IL-13 than the WT-PPE group (**Figure 4B**, panels a and f). In addition, there were no significant differences between the WT-PPE and *Has2* ^+/−^-PPE mice in terms of the levels of IL-1β, IL-6, or IL-17, all of which are associated with neutrophilic inflammation (**Figure 4A**, panels d–f). Based on these results, *Has2* attenuation induced severe neutrophilic airway inflammation due to increased G-CSF after PPE stimulation.

### Elastase stimulation induced high-molecular-weight hyaluronan via *Has2*, and the TGF-β1 pathway activation

**Figure 5:**
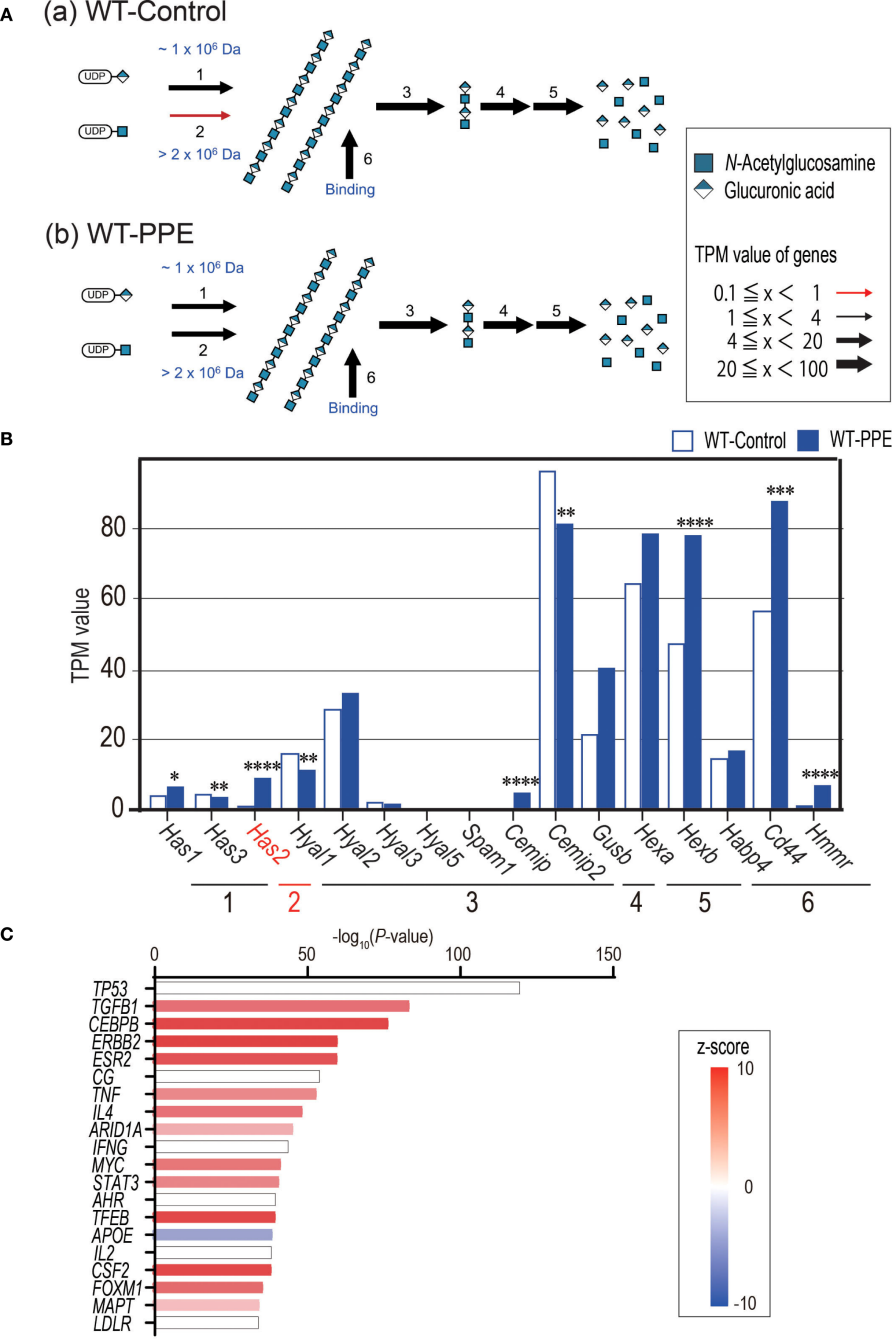
PPE administration affects the HMW-HA synthesis and activates the TGF-b1-related pathway. **(A)** Biosynthesis and catabolism of hyaluronan pathways in the lungs of WT-Control mice **(A)** and WT-PPE mice **(B)** were visualized using GlycoMaple. **(B)** The TPM value of hyaluronan pathway related genes between the lungs of WT-Control and WT-PPE mice (n = 7). *P < 0.05, **P < 0.01, ***P < 0.001, ****P < 0.0001 relative to the WT-Control mice. **(C)** The top 20 significant genes detected via upstream analysis of the DEGs in the lungs of WT-Control and WT-PPE mice (n = 7). DEGs, differentially expressed genes.

RNA-seq was conducted to elucidate the alterations in gene expression that occurred 4 days after the administration of PPE in the WT and *Has2* ^+/−^ mice. Initially, a comprehensive analysis of the glycosylation pathway was conducted to visualize the impact of PPE administration on the hyaluronic acid synthesis pathway using Glycomaple. The results of the analysis, which were based on the transfer product number per million (TPM), showed that the synthesis pathway of HMW-HA was suppressed in the non-stimulated state (**Figure 5A**, panel a). However, it was activated after the administration of PPE (**Figure 5A**, panel b). Among the 16 genes implicated in the biosynthesis and catabolism of hyaluronan pathway that were examined, only *Has2* has been implicated in the synthesis of HMW-HA (**Figures 5A, B**). The WT mice exhibited an increase in the *Has2* gene expression after the administration of PPE (**Figure 5B**). However, the *Has2* gene expression did not differ between the WT-PPE and *Has2* ^+/−^ -PPE mice (**Supplementary Figure 5**). The generation of *Has2*-deficient mice was established with 60-codon deletion in exon 4. This short deletion could potentially explain the absence of differences in the gene expression that were not detected via RNA-seq. Further, based on the results of an upstream analysis using the Ingenuity Pathway Analysis software, the response to the administration of PPE was significantly regulated by TGFB1 gene activation (**Figure 5C**, **Supplementary Table 1**). These findings indicate that the elastase-induced emphysema model led to the production of HMW-HA via *Has2*, which is then regulated via the TGF-β1 pathway.

### *Has2* attenuation affected inflammatory phenotype, macrophage differentiation, and impaired the TGF-β-related signaling

**Figure 6:**
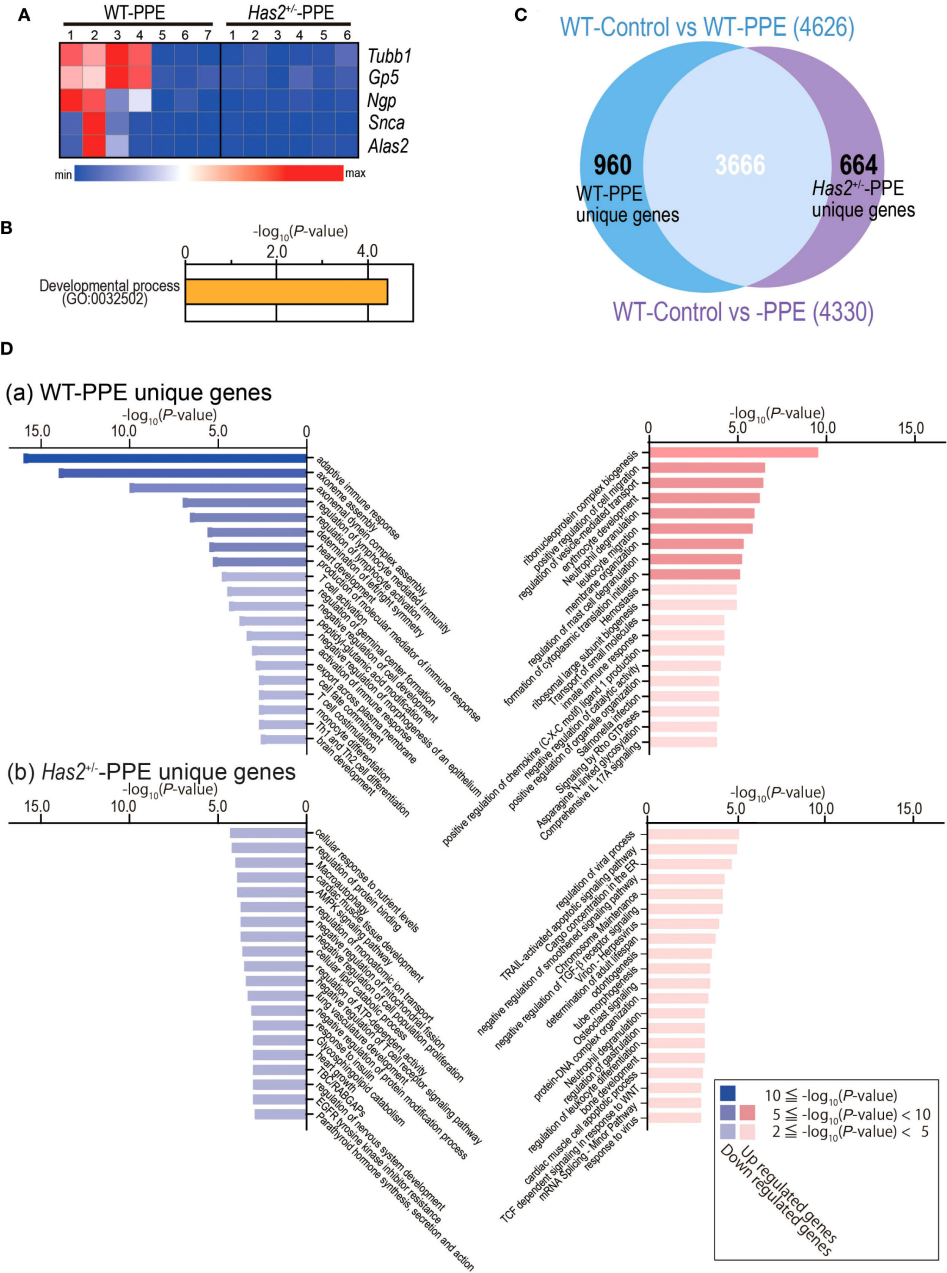
DEG analysis between the lungs of the WT-Control, WT-PPE, and *Has2* ^+/−^-PPE mice. **(A)** Heatmap of the DEGs in the lungs of WT-PPE and *Has2* ^+/−^-PPE mice (n = 6–7, cutoff: adjusted P < 0.01; log_2_ fold change > 1.2.). Hierarchical clustering was performed based on the mean log_2_ fold change. **(B)** Significant biological process terms detected via GO analysis in WT-PPE and *Has2* ^+/−^-PPE mice. **(C)** Identification of the unique DEGs between WT-Control vs. WT-PPE mice and WT-Control vs. *Has2* ^+/−^-PPE mice using the Venn diagram. **(D)** The top 20 significant terms detected via GO analysis among the unique genes in the WT-PPE mice (a) and *Has2* ^+/−^-PPE mice (b).

Five genes had significantly different expressions in the lungs of WT-PPE mice and *Has2* ^+/−^-PPE mice (**Figure 6A**). GO analyses identified myeloid cell differentiation (GO: 0030099) (**Figure 6B**). Next, our analysis focused on specifically altered genes in WT-PPE and *Has2* ^+/−^-PPE mice compared with WT-Control mice (**Figure 6C**). In total, 960 genes were uniquely altered in the WT-PPE mice (WT-PPE unique genes, **Figure 6C**). Meanwhile, 664 genes were uniquely altered in the *Has2* ^+/−^-PPE mice (*Has2* ^+/−^-PPE unique genes, **Figure 6C**). GO analyses revealed several significant biological processes, including ribonucleoprotein complex biogenesis (GO: 0022613), neutrophil degranulation (R-MMU-6798695), leukocyte migration (GO: 0050900), innate immune response (GO: 0045087), and comprehensive IL-17A signaling (WP5242) among the uniquely upregulated genes in WT-PPE (**Figure 6D**, panel a, right lane, and **Supplementary Table 2**). Conversely, the pathways associated with adaptive immune response (GO: 0002250, GO: 0002706, GO: 0051249, GO: 0042110, GO: 0002253, GO: 0031295, and mmu04658) was identified among the uniquely downregulated genes in the WT-PPE mice (**Figure 6D**, panel a, left lane, **Supplementary Table 3**). These results might be related to the increase in the lymphocyte counts in the BALF of the WT-PPE group during the acute phase, and the significant increase in the neutrophil counts in the *Has2* ^+/−^-PPE group. The pathways associated with ciliary movement (GO: 0035082 and GO: 0070286) and heart development (GO: 0007507) were also identified among the uniquely downregulated genes in the WT-PPE mice (**Figure 6D**, panel a, left lane, **Supplementary Table 3**). Further, the pathways related to cargo concentration in ER (R-MMU-5694530) and the negative regulation of transforming growth factor beta receptor signaling pathway (GO: 0030512) were detected in the uniquely upregulated genes in the *Has2* ^+/−^-PPE mice (**Figure 6D**, panel b, right lane, **Supplementary Table 4**). These pathways, detected in *Has2* ^+/−^-PPE unique genes, are associated with HA or *Has2* (14, 19).

**Figure 7:**
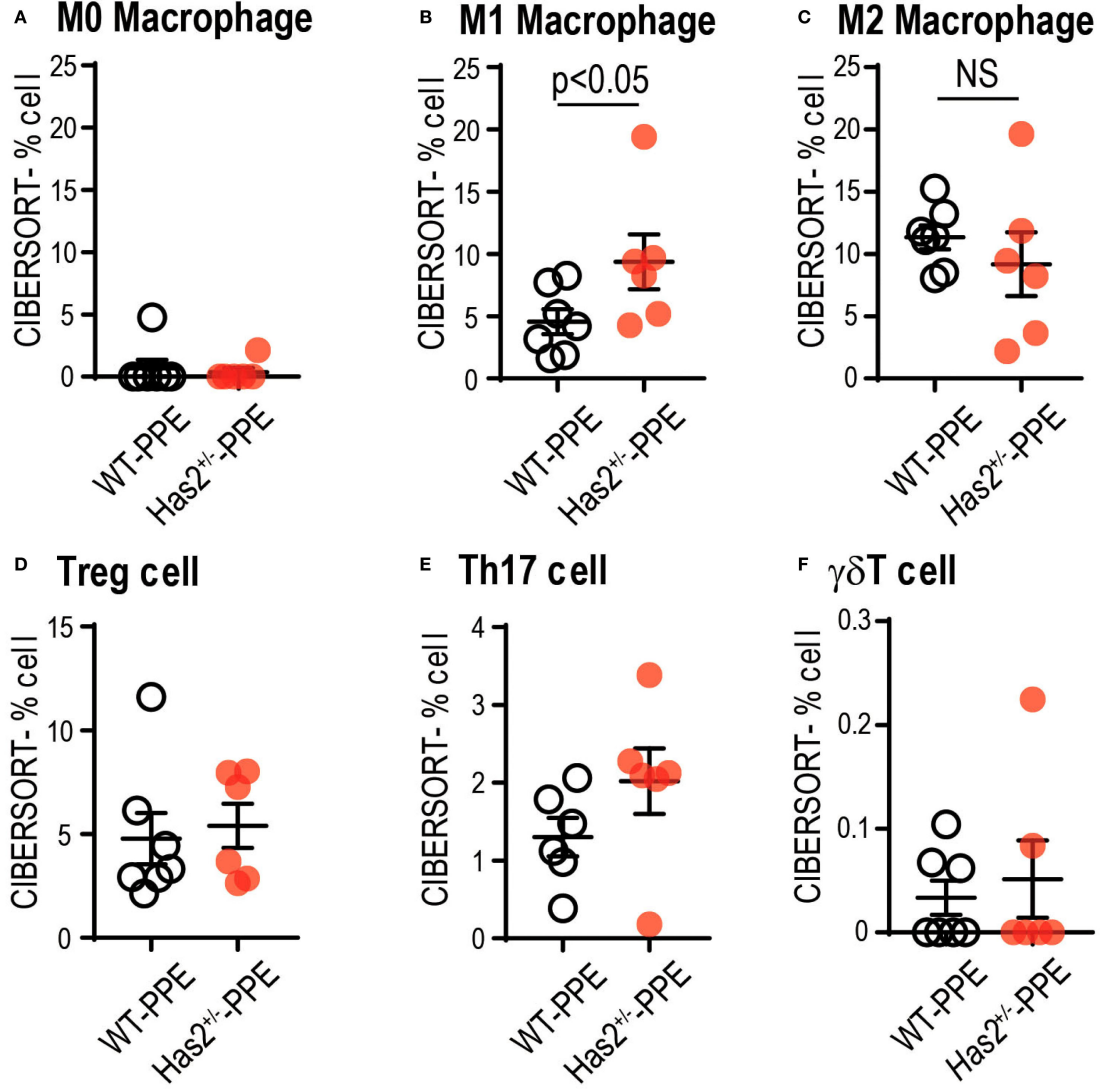
Attenuation of Has2 affected macrophage differentiation. Semi-quantitative evaluation of immune cell infiltrates after PPE stimulation, as determined via CIBERSORT analysis on whole-lung RNA-seq data (n = 6–7). **(A)** M0 Macrophage. **(B)** M1 Macrophage. **(C)** M2 Macrophage. **(D)** Treg cell. **(E)** Th17 cell. **(F)** γδT cell. All samples were obtained 4 days after the PPE stimulation. Statistical significance was determined using the Mann–Whitney U test. Horizontal bars indicate direct statistical comparisons between WT and *Has2* ^+/−^ mice. NS, not significant.

To understand the significant changes in immune cell fractions and cytokines in the lungs of WT-PPE and *Has2* ^+/−^-PPE mice, a CIBERSORT analysis was performed. In this analysis, the proportion of M1 macrophage cells was significantly high in the *Has2* ^+/−^-PPE mice (**Figure 7**). However, there were no significant differences in the proportion of Treg cells, Th17 cells, and γδT cells affecting the production of TGF-β or IL-17 between the two groups (**Figure 7**). Based on these results, the differentiation of M1 macrophages in the *Has2* ^+/−^-PPE group may be affected by the dysfunction of *Has2* under these conditions.

## Discussion

The protease–antiprotease imbalance hypothesis was proposed after the discovery of alpha-1 antitrypsin deficiency, a disease susceptibility gene for COPD (20, 21). Currently, this hypothesis is applicable to COPD that is not associated with alpha-1 antitrypsin deficiency (21). A previous study reported that smoking also causes a reduction in the ability of alpha-1 protein to inhibit elastase activity (21). HA is relevant the vulnerability of matrix elastin to the effects of elastases (21, 22). The loss of HA was observed in human emphysema lungs (23, 24). Meanwhile, aerosolized HA significantly reduced elastase- or cigarette smoke-induced airspace enlargement (21, 25). Based on these data, the design of the current study was established.

To the best of our knowledge, this study first report that the attenuation of *Has2* mRNA affected the severity of PPE-induced pulmonary emphysema and airway inflammation in the *Has2^+/−^* mice. A quantitative analysis using MLI revealed that the *Has2^+/−^* mice exhibited more severe emphysema at least 1 day after the administration of PPE compared with the WT mice (**Figure 1C**). *Has2^+/−^* mice showed a significant increase and prolongation in the number of BALF neutrophils after PPE stimulation (**Figure 2D**). Consistent with previous studies, an increase in the lymphocyte and eosinophil counts was observed during the acute phase in the WT mice (26, 27). However, the *Has2^+/−^* mice had a lower increase in the lymphocyte and eosinophil counts than the WT mice. The *Has2^+/−^* mice had significantly lower lymphocyte counts on day 1 after the administration of PPE than the WT mice (**Figure 2C**). This phenotypic difference may be attributed to the significantly higher number of BALF neutrophils compared with other cells in the *Has2^+/−^*-PPE mice.

In the cytokine and chemokine analyses, a significantly increase in the G-CSF levels was observed in the lungs of the *Has2^+/−^* mice on day 1 after the administration of PPE (**Figure 4A**, panel a). G-CSF is an important factor in the survival, proliferation, and differentiation of progenitor cells that differentiate into neutrophils (28). Previous studies have reported an association between genetic variation in the G-CSF and a decline in the forced expiratory volume in 1 second (FEV_1_) in smokers (29), and the elevated levels of G-CSF in the BALF of patients with COPD (30). In addition, high serum G-CSF levels promote COPD exacerbations characterized by neutrophilic inflammation with underlying bacterial dysbiosis (31). Further, the deletion of G-CSF reduces airway inflammation, lung tissue destruction, and the severity of comorbidities in mice (30). Based on these results, the increase in the G-CSF levels associated with *Has2* dysfunction worsens emphysema formation and airway inflammation during the acute phase after PPE administration in the *Has2^+/−^*-KO mice. Conversely, there were no significant increases in the levels of other neutrophil- or Th1-related cytokines in the lung homogenates of the *Has2^+/−^*-PPE mice (**Figure 4**, **Supplementaary Figures 2, 3**). In addition, the expression of Th2-type cytokines, including IL-4, IL-5, and IL-13, decreased in the lung homogenates of the *Has2^+/−^*-PPE mice compared with the WT mice (**Figure 4B**). Similar results were observed in our previous chronic Ovalbumin (OVA)-stimulated asthma model (14). Based on these findings, the exacerbation of airway inflammation due to *Has2* dysfunction is influenced by a pathway that is different from the Th1/Th2 imbalance pathway.

HA, CD44, and TGF-β1 are involved in the resolution of acute inflammation of tissue damage occurs (32–34). In the bleomycin-induced acute lung injury model, 75% of CD44-deficient mice die within a 2-week period. The underlying cause of this condition is believed to be abnormalities in the inflammatory resolution mechanism, characterized by low TGF-β1 activity, impaired removal of apoptotic neutrophils, and accumulation of HA (32). Moreover, TGF-β has a protective role for airway inflammation and AHR (35, 36). However, it exacerbates airway fibrosis and airway muscle hyperplasia (37). In the current study, RNA-seq analysis revealed high levels of *Has2* mRNA expression in the lungs 4 days after the administration of PPE (**Figure 5B**). Using GlycoMaple to elucidate the alterations in the synthetic metabolic pathway of HA, an enhancement in the HMW-HA synthetic pathway was observed following the administration of PPE (**Figure 5A**). An upstream analysis revealed that the differentially expressed genes that underwent alterations subsequent to the administration of PPE in the WT mouse lungs were under the regulatory influence of TGF-β1 (**Figure 5C**). Further, GO analysis revealed that the negative regulation of the TGF-β receptor signaling pathway was significantly enriched among the uniquely upregulated genes in the *Has2* ^+/−^-PPE mice (**Figure 6D**, panel b, right lane, **Supplementary Table 4**). A statistically significant difference in the TGF-β concentrations was observed between the WT mice and *Has2^+/−^* mice groups only 21 days after the administration of PPE (**Figure 4**). However, the *Has2^+/−^* mice were more likely to have lower protein-adjusted TGF-β concentrations. Based on these results suggest that *Has2* inhibition leads to the sustained suppression of HA-binding protein expression and TGF-β signaling.

Regardless of the TGF-β pathway results, the GO analysis results using the unique genes in the WT-PPE or *Has2* ^+/−^-PPE mice are supported by the findings of this study. Several significant biological processes, which are related to neutrophilic airway inflammation, were observed among the uniquely upregulated genes in the WT-PPE mice (**Figure 6D**, panel a, right lane, **Supplementary Table 2**). Meanwhile, the pathways associated with the adaptive immune response were identified among the unique downregulated genes in the WT-PPE mice (**Figure 6D**, panel a, left lane, **Supplementary Table 3**). These results are consistent with the BALF results (**Figure 2**). In particular, the airway inflammation phenotype in the *Has2* ^+/−^-PPE mice is predominantly neutrophil-dominant, with lymphocyte-mediated airway inflammation being relatively suppressed compared with that in the WT mice. In our previous RNA-seq analysis conducted under chronic OVA stimulation, the downregulation of EIF2 signaling pathways, which are associated with ER stress response, and the TGF-β signaling pathways, and Th17 bias were observed in the BALB/c background *Has2* ^+/−^ mice (14). In the current study, RNA-seq analysis identified comprehensive IL-17A signaling among the uniquely upregulated genes in the WT-PPE mice. Further, cargo concentration in the ER was detected among the uniquely upregulated genes in the *Has2* ^+/−^-PPE mice (**Figure 6D**, panel b, right lane, **Supplementary Table 4**). However, the roles of IL-17 and endoplasmic reticulum stress in this process are not yet completely understood. This may be attributed to the fact that PPE is a more potent stimulus than OVA in inducing G-CSF and neutrophilic inflammation, which complicates the detection of CD4+ T cells compared with OVA. Furthermore, previous studies have reported that C57BL/6 mice exhibit weaker increases in IL-17 than BALB/c mice in PPE administration models (24). Therefore, although significant increases in IL-6 and G-CSF were observed in the lung homogenate samples from this experiment, changes in IL-17 were likely difficult to detect.

The findings of this study do not provide a comprehensive explanation for the observed association between *Has2* dysfunction and the subsequent development of acute-phase G-CSF and neutrophil increase, accompanied by severe emphysema formation. However, based on previous research results, there are several hypotheses that must be considered. In a bleomycin-induced pulmonary fibrosis model, *Has2* induces and regulates cellular senescence in fibroblasts via the p27-CDK2-SKP2 pathway (38). Further, recent studies have revealed that fibroblasts responsive to FGF promote the production of G-CSF, HA secretion, and neutrophilic inflammation, thereby contributing to steroid-resistant airway inflammation in patients with severe asthma (39). In light of the aforementioned data, it can be hypothesized that *Has2* dysfunction in fibroblasts may influence G-CSF production and HA secretion, thereby exacerbating the pathogenesis of inflammatory lung diseases. Considering that the proportion of M1 macrophages in the *Has2* ^+/−^ mice was significantly higher than that of the WT mice in the CIBERSORT analysis (**Figure 7**) and that alveolar destruction progressed after PPE administration, it can be hypothesized that *Has2* expression abnormalities directly affect M1 macrophages and epithelial cells. Live *E. coli* instilled into the airways of mice induced rapid and selective increases in the mRNA expression of *Has1* and *Has2* in the whole lung (40).Primary cultures of murine airway epithelial cells showed significant increases in the *Has2* expression (40). However, the upregulation of *Has1* expression, but not *Has2* expression, in the macrophages was found to be a selective response to LPS, the M1 classical activation via TLR4 *in vitro* (40). These results indicate that the *Has2* expression in epithelial cells and fibroblasts may directly influence this disease state.

The current study had several limitations that should be considered before translating the findings into therapies for airway diseases. For instance, because the present study used lung bulk RNA sequencing, cell-type-specific data could not be obtained. Further research is needed to validate these mechanisms using methods such as single-cell RNA sequencing, spatial transcriptomics, and lineage-specific *Has2* knockouts in fibroblast, epithelial, and macrophage cells. Additionally, the airway glycan ligand that acts on CD44 or regulates G-CSF secretion may have decreased in the *Has2^+/−^* mice. However, this decrease has not been confirmed. Therefore, future experiments should be performed to confirm treatment efficacy. In particular, whether the supply of HAS2 enzymes, HMW-HA, and anti-G-CSF Ab into the airway inhibits lung neutrophilic airway inflammation and emphysema should be examined. Furthermore, determining whether the administration of TGF-β signaling modulators exacerbates COPD pathology could be useful in evaluating potential therapeutic targets. Recent clinical studies have shown that the inhalation of HMW-HA decreases the duration of respiratory failure and the need for noninvasive ventilation therapy in patients experiencing acute exacerbation of COPD (41). Further, the inhibition of G-CSF receptors reduces neutrophilic inflammation, mucosal injury, and airways fibrosis (42, 43). These results show the clinical efficacy of treatments targeting hyaluronic acid abnormalities and support our results. Additionally, some experiments, such as cytokine assays and RNA-seq, have small sample sizes, which limits their power and generalizability. By increasing the sample size, it may be possible to detect cytokine changes and DEGs under *Has2* functional suppression that were not detected in this study. Previous studies have reported that the progression of emphysematous changes persists for up to two to three months (26). A longer observation period is required because TGF-β is significantly reduced in *Has2* ^+/-^ mice 21 days after PPE administration. However, this study did not evaluate TGF-β-related structural changes including small airway remodeling such as airway wall thickness, collagen deposition, airway smooth muscle hypertrophy. Further investigation, including a morphological assessment of small airway remodeling at late onset, is necessary to elucidate the characteristic phenotypes associated with the mechanistic relationship between TGF-β and extracellular matrix homeostasis under HA dysfunction. Furthermore, this study did not validate the use of data from COPD patients or human lung tissue. Further validation using human specimens is necessary for clinical application. For instance, evaluating the activity of HAS2, the expression levels and fractions of HMW-HA and LMW-HA, and the concentrations of G-CSF and TGF-β in lung specimens from COPD patients, as well as examining their correlation with FEV_1_ decline, the frequency of acute exacerbations, and prognosis, is expected to enhance their clinical value.

Nevertheless, the observation that *Has2^+/−^* mice exhibited more severe emphysema progression, neutrophilic airway inflammation accompanied by a significant increase in the G-CSF levels, and reduced TGF-β levels in the lungs strongly indicate that low *Has2* levels impair extracellular matrix homeostasis for controlling PPE-induced lung injury. Further, disorders associated with reduced HAS2 function in the lungs may result in persistent airway inflammation and remodeling. The serum HA concentrations of patients with COPD increased during exacerbations and remained high. Thus, serum HA can be an independent predictor of overall survival (44). Elucidating the effects of HAS2 and HA could help identify patients with COPD who have poor prognosis. Moreover, this research could support the efficacy of HA inhalation therapy, anti-G-CSF antibody therapy, and G-CSF receptor-targeted antagonistic therapy in patients with COPD. Considering these factors, the results of this study could help improve the mortality rates of COPD patients.

## Data Availability

The datasets presented in this study can be found in online repositories. The names of the repository/repositories and accession number(s) can be found below: GSE304417 (GEO). https://www.ncbi.nlm.nih.gov/geo/query/acc.cgi?acc=GSE304417.
