# Supplementary material for: Hyaluronic acid synthase 2 dysfunction exacerbates elastase-induced neutrophilic airway inflammation and emphysema in mice
Source: Front Immunol. 2025 Oct 14;16:1683385. doi: 10.3389/fimmu.2025.1683385 (PMC12577564; doi:10.3389/fimmu.2025.1683385)
Supplement: Supplementary file 1 [file DataSheet1.docx]

Supplementary Material

**
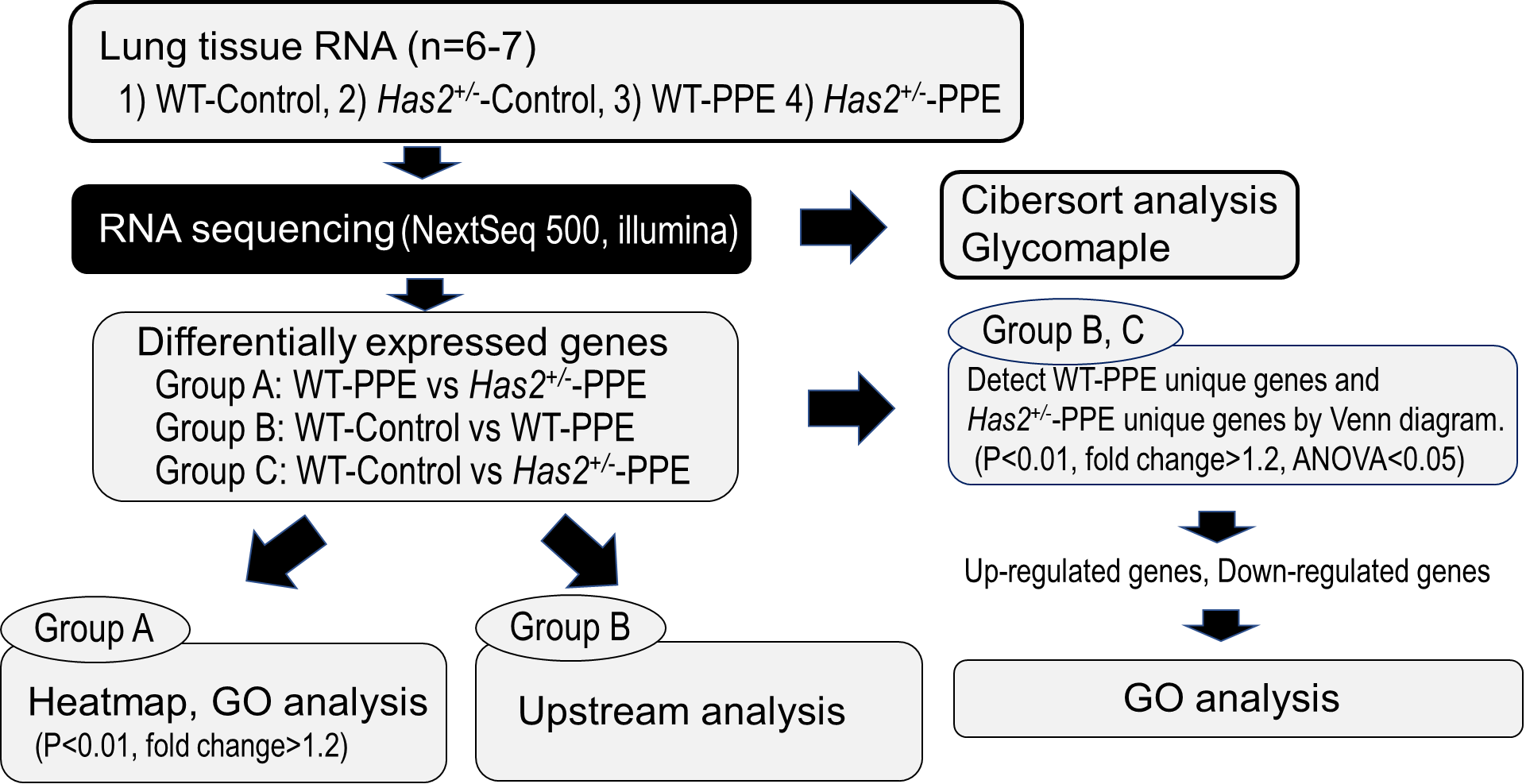
**

**Supplementary figure 1. Schematic workflow for the analysis of gene lists generated from the transcriptome profiling experiments.** Gene lists represent gene products significantly upregulated and downregulated in the lungs under the following three conditions: (group A) WT-PPE mice compared with *Has2*^+/−^-PPE mice, (group B) WT-Control mice compared with WT-PPE mice, and (group C) WT-saline mice compared with *Has2*^+/−^-PPE mice. Lists of DEGs were prepared and analyzed by following software: Cibersort (<https://cibersort.stanford.edu/>), Glycomaple ((https://glycosmos.org/glycomaple/Human), Morpheus (<https://software.broadinstitute.org/morpheus/>), Metascape (<http://metascape.org>), and ingenuity pathways analysis (IPA) software. GO, gene ontology.


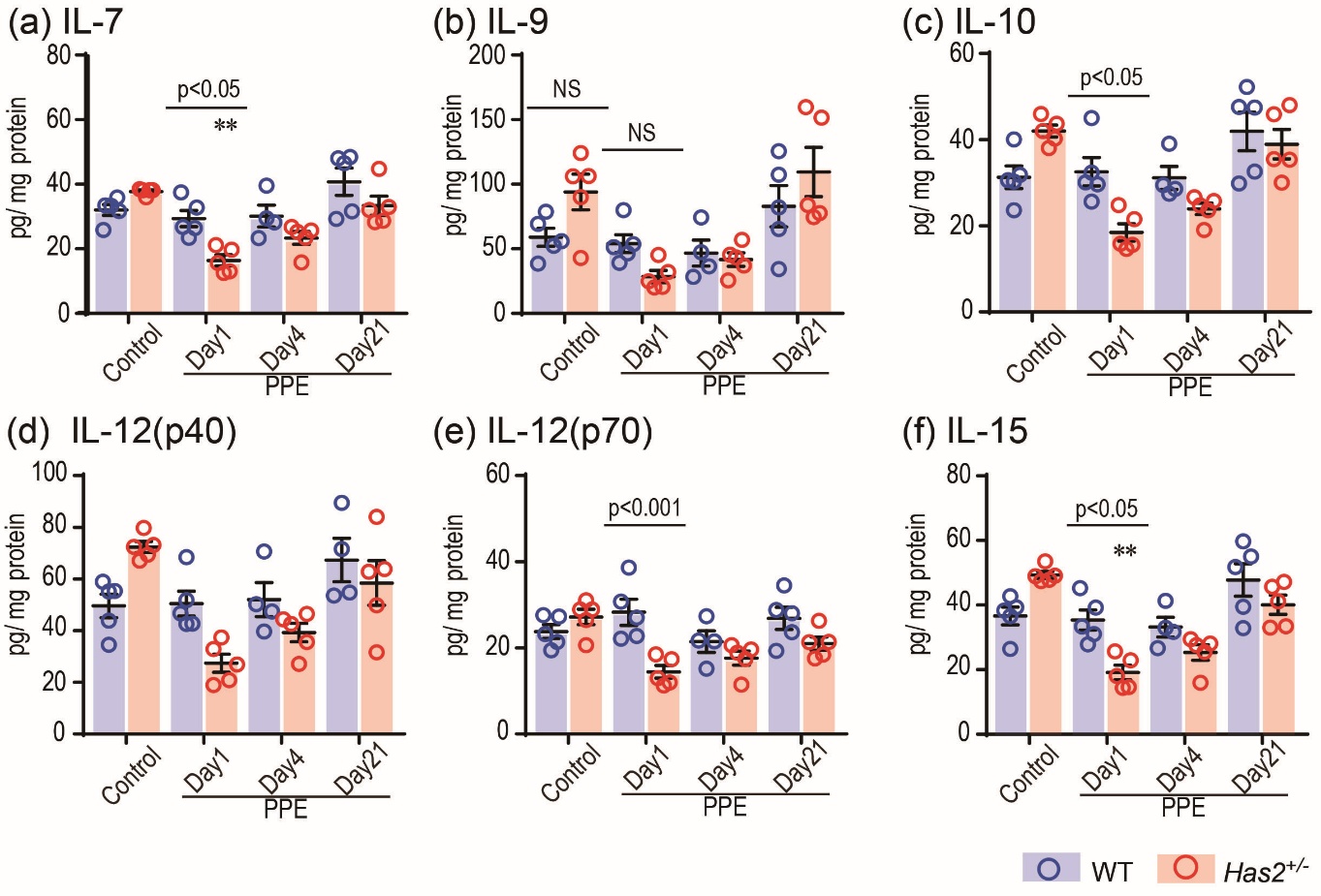


**Supplementary figure 2. Effect of *Has2* attenuation on the cytokine levels in lung homogenate.** Protein adjusted levels of inflammatory chemokines in lung homogenates (n = 4–5). Statistical significance was determined using Tukey’s multiple comparison test. ** P < 0.01 relative to the WT-control mice. Horizontal bars indicate direct statistical comparisons between WT and *Has2*^+/−^ mice. NS, not significant.


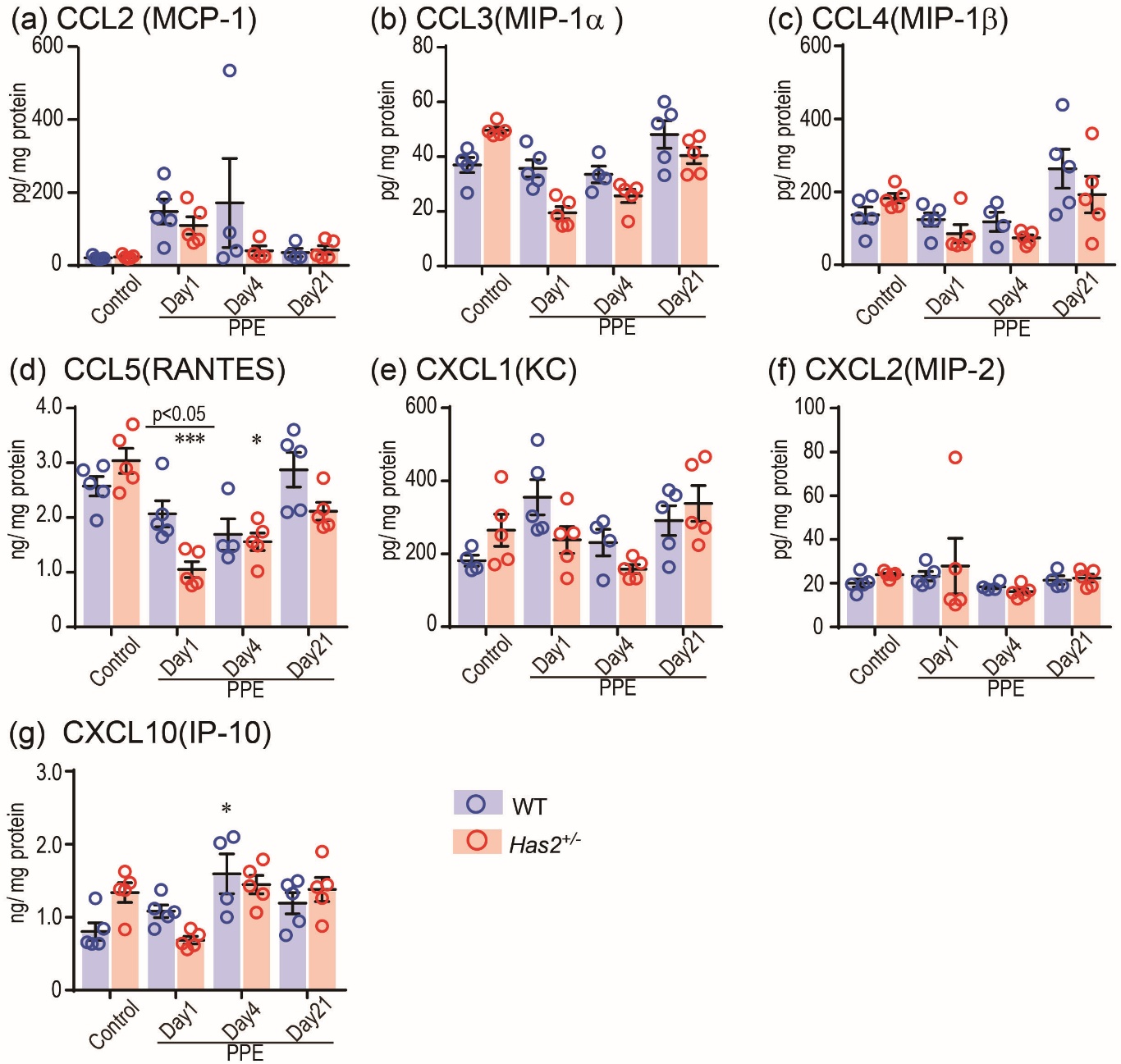


**Supplementary figure 3. Effect of *Has2* attenuation on the chemokine levels in lung homogenate.** Protein adjusted levels of inflammatory chemokines in lung homogenates (n = 4–5). Statistical significance was determined using Tukey’s multiple comparison test. *P < 0.05 and ***P < 0.001 relative to the WT-control mice. Horizontal bars indicate direct statistical comparisons between WT and *Has2*^+/−^ mice.


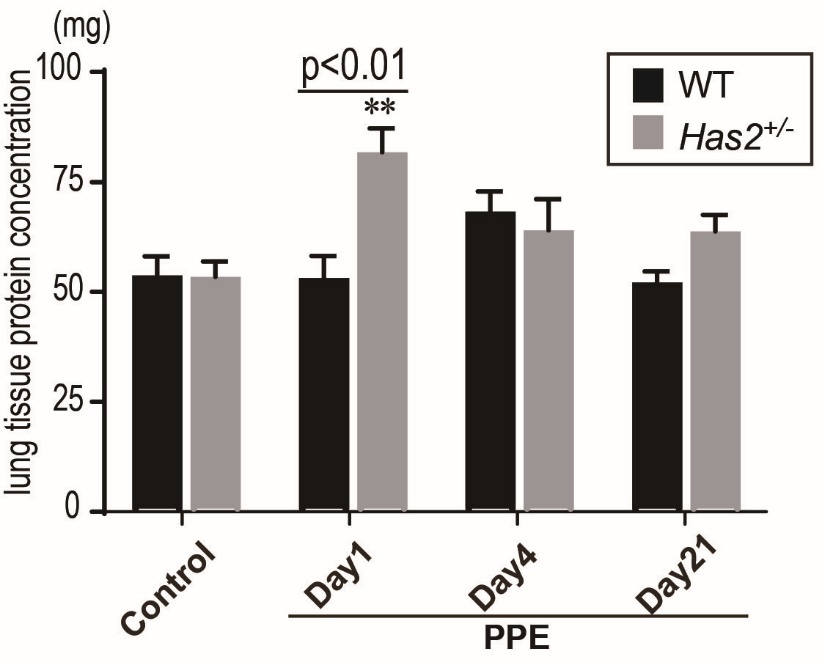


**Supplementary figure 4. Effect of *Has2* attenuation on the chemokine levels in lung homogenate.** Protein levels in lung homogenates (n = 4–5). Statistical significance was determined using Tukey’s multiple comparison test. ** P < 0.01 relative to the WT-control mice. Horizontal bars indicate direct statistical comparisons between WT and *Has2*^+/−^ mice.

**
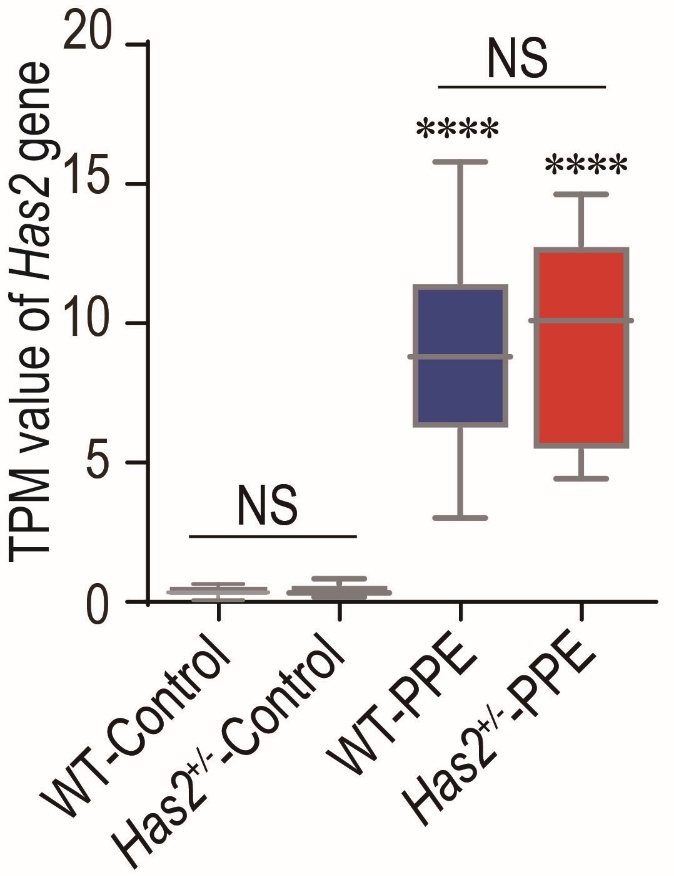
**

**Supplementary figure 5. TPM value of *Has2* gene between the lungs of WT-control and WT-PPE mice.** The TPM value of *Has2* gene between the lungs of WT-control and WT-PPE mice (n = 6–7), **** P < 0.0001 relative to the WT-control mice. NS, not significant.

**Supplementary Table 1.** Top 20 significant molecule detected using upstream analysis with IPA in DEGs between WT-control and WT-PPE mice.

| Upstream Regulator | Molecule Type | Predicted Activation State | Activation z-score | p-value of overlap |
| --- | --- | --- | --- | --- |
| *TP53* | transcription regulator |  | -0.983 | 4.79E-120 |
| *TGFB1* | growth factor | Activated | 5.433 | 6.46E-84 |
| *CEBPB* | transcription regulator | Activated | 7.749 | 5.58E-77 |
| *ERBB2* | kinase | Activated | 7.993 | 1.90E-60 |
| *ESR2* | ligand-dependent nuclear receptor | Activated | 7.045 | 2.38E-60 |
| *CG* | complex |  | -0.25 | 1.65E-54 |
| *TNF* | cytokine | Activated | 4.262 | 1.48E-53 |
| *IL4* | cytokine | Activated | 5.332 | 6.54E-49 |
| *ARID1A* | transcription regulator | Activated | 2.706 | 8.10E-46 |
| *IFNG* | cytokine |  | 1.331 | 3.03E-44 |
| *MYC* | transcription regulator | Activated | 5.137 | 6.83E-42 |
| *STAT3* | transcription regulator | Activated | 4.432 | 2.98E-41 |
| *AHR* | ligand-dependent nuclear receptor |  | -1.764 | 2.20E-40 |
| *TFEB* | transcription regulator | Activated | 7.652 | 4.00E-40 |
| *APOE* | transporter | Inhibited | -3.109 | 4.29E-39 |
| *IL2* | cytokine |  | 0.24 | 6.53E-39 |
| *CSF2* | cytokine | Activated | 7.648 | 8.63E-39 |
| *FOXM1* | transcription regulator | Activated | 5.743 | 5.18E-36 |
| *MAPT* | other | Activated | 2.086 | 7.17E-35 |
| *LDLR* | transporter |  | 0.401 | 8.58E-35 |

**Supplementary Table 2.** Top20 Significant terms detected using GO analysis with metascape in WT-PPE unique up regulated differentially expressed genes.

| **Up regulated gene** | | | | |
| --- | --- | --- | --- | --- |
| GO Term | | -log_10_(*p*-value) | InTerm_InList | Symbols |
| GO:0022613 | ribonucleoprotein complex biogenesis | 9.50 | 31/428 | *Eif3a\|Utp4\|Xrcc5\|Exosc9\|Rpp30\|Eif3d\|Ddx21\|Tssc4\|Noc2l\|Rrs1\|Nip7\|Tma16\|Rsl1d1\|Riok3\|Rrp15\|Nop9\|Mak16\|Zfp593\|Gar1\|Nol11\|Wdr77\|Pwp1\|Pwp2\|Prmt7\|Utp18\|Eif5\|Dcaf13\|Nol6\|Heatr3\|Ppan\|Urb2* |
| GO:0030335 | positive regulation of cell migration | 6.50 | 32/609 | *Alox12\|Anxa3\|Cdc42\|Cxcr2\|Creb3\|Csf1\|Epha2\|Fgr\|Gab2\|Gpi1\|Ccn1\|Il1b\|Ilk\|Itga2b\|Itgb1\|Lbp\|Mmp3\|Mmp9\|Fam89b\|Ptp4a1\|Sphk1\|Stat3\|Trip6\|Vegfc\|Myadm\|Nus1\|Ccl24\|Trpv4\|Smoc2\|Rras2\|Rhoj\|Tirap* |
| GO:0060627 | regulation of vesicle-mediated transport | 6.40 | 32/613 | *Arf1\|Cd84\|Cdc42\|Doc2b\|Epha3\|Fgr\|Flot1\|Gab2\|Gata1\|Itgb1\|Lbp\|Msn\|Rab4b\|Snca\|Sphk1\|Stxbp2\|Sdc4\|Snx9\|Chmp2a\|Snx4\|Scfd1\|Mctp1\|Siglece\|Hamp\|Pls3\|Bmp2k\|Mical1\|Cd300a\|Arhgap1\|Rab8b\|Dnajc13\|Abca13* |
| GO:0048821 | erythrocyte development | 6.20 | 9/52 | *Alas2\|Bpgm\|Epb42\|Gata1\|Hba-a1\|Rhag\|Slc4a1\|Hba-a2\|Heatr3* |
| R-MMU-6798695 | Neutrophil degranulation | 5.90 | 28/521 | *Atp6v0a1\|Cd33\|Cd53\|Cxcr2\|Camp\|Fgr\|Gpi1\|Mmp9\|Psmd7\|Lilra6\|Rab4b\|S100a8\|S100a9\|Cyfip1\|Serpinb6a\|Xrcc5\|Actr2\|Pgm1\|Commd9\|Siglece\|Plekho2\|Cyb5r3\|Cd300a\|Actr1b\|Fcgr4\|Abca13\|Dynlt1a\|Hbb-bs* |
| GO:0050900 | leukocyte migration | 5.80 | 18/247 | *Add2\|Cdc42\|Cxcr2\|Csf1\|Csf3r\|Eps8\|Il17ra\|Il1b\|Itgb1\|Lbp\|Mmp9\|Msn\|S100a8\|S100a9\|Ccl24\|Gpr35\|Cklf\|Retnlg* |
| GO:0061024 | membrane organization | 5.30 | 32/692 | *Slc25a4\|Arf1\|Cdc42\|Flot1\|Folr1\|Gnai3\|Hdac3\|Ier3\|Itgb1\|Ndrg1\|Abcb4\|Sar1a\|Slc4a1\|Snca\|Spta1\|Stat3\|Cln8\|Myadm\|Mymk\|Snx9\|Get4\|Apoo\|Chmp2a\|Tmem43\|Immt\|Siglece\|Exoc5\|Rab8b\|Mmgt1\|Rft1\|Wdr83os\|Dynlt1a* |
| GO:0043304 | regulation of mast cell degranulation | 5.20 | 7/37 | *Cd84\|Fgr\|Gab2\|Gata1\|Stxbp2\|Snx4\|Cd300a* |
| GO:0001732 | formation of cytoplasmic translation initiation complex | 5.10 | 3/3 | *Eif3a\|Eif3d\|Eif5* |
| R-MMU-109582 | Hemostasis | 4.90 | 25/492 | *Cd84\|Cdc42\|F5\|Fgr\|Gata1\|Gnai3\|Gnb2\|Gng12\|Gp1bb\|Itga2b\|Itgb1\|Irag1\|Nfe2\|Serpinb2\|Prkar2b\|Shc1\|Serpinb6a\|Stxbp2\|Sdc4\|Tuba4a\|Vegfc\|Ywhaz\|Sccpdh\|Abcc4\|Tubb1* |
| GO:0042273 | ribosomal large subunit biogenesis | 4.90 | 9/75 | *Noc2l\|Rrs1\|Nip7\|Tma16\|Rsl1d1\|Rrp15\|Mak16\|Heatr3\|Ppan* |
| R-MMU-382551 | Transport of small molecules | 4.20 | 28/645 | *Add2\|Slc25a4\|Atp1a1\|Atp1b3\|Atp6v0a1\|Phb2\|Gnb2\|Gng12\|Psmd7\|Abcb4\|Prkar2b\|Psma3\|Psme3\|Rhag\|Slc4a1\|Arl2\|Angptl4\|Trpv4\|Stoml2\|Apobr\|Soat2\|Abcc4\|Ano2\|Erlin2\|Nceh1\|Slc7a6\|Parl\|Hbb-bs* |
| GO:0045087 | innate immune response | 4.20 | 31/751 | *Phb2\|Cd84\|Cdc42\|Camp\|Csf1\|Fgr\|Il17ra\|Irak1\|Lbp\|Mmp3\|Padi4\|Trim10\|S100a8\|S100a9\|Snca\|Stxbp2\|Xrcc5\|Ddx21\|Ccl24\|Cdc42ep4\|Ube2l6\|Actr2\|Riok3\|Polr3k\|Plekhm2\|Tmem43\|Tirap\|Tlr8\|Ifitm6\|Fcgr4\|Nlrx1* |
| GO:2000340 | positive regulation of chemokine (C-X-C motif) ligand 1 production | 4.20 | 3/5 | *Il17ra\|Trpv4\|Tirap* |
| GO:0043086 | negative regulation of catalytic activity | 4.00 | 26/597 | *Anxa8\|Prdx3\|Cast\|Gpx1\|Il1b\|Mmp9\|Ngp\|Serpinb2\|Prkar2a\|Prkar2b\|Slc4a1\|Snca\|Serpinb9b\|Serpinb6a\|Stfa3\|Cln8\|Arl2\|Angptl4\|Nolc1\|Mad2l2\|Camk2n2\|Spred1\|Mical1\|Wnt9a\|Cd300a\|Zfyve28* |
| GO:0010638 | positive regulation of organelle organization | 3.90 | 24/532 | *Slc25a4\|Cct5\|Cdc42\|Doc2b\|Mapre1\|Il1b\|Mmp9\|Msn\|Cyfip1\|Snca\|Sphk1\|Sdc4\|Xrcc5\|Ccl24\|Arl2\|Cdc42ep4\|Trpv4\|Vps35\|Snx9\|Marchf5\|Snx4\|Nabp2\|Ddhd1\|Ino80c* |
| mmu05132 | Salmonella infection - Mus musculus (house mouse) | 3.90 | 15/253 | *Arf1\|Cdc42\|Il1b\|Irak1\|Cyfip1\|Tuba4a\|Snx9\|Actr2\|Plekhm2\|Rhoj\|Exoc5\|Tirap\|Actr1b\|Tubb1\|Dynlt1a* |
| R-MMU-194315 | Signaling by Rho GTPases | 3.90 | 25/572 | *Arhgdib\|Cdc42\|Mapre1\|Epha2\|Flot1\|Flot2\|Itgb1\|Ncf2\|S100a8\|S100a9\|Cyfip1\|Tuba4a\|Ywhaz\|Cops4\|Zwint\|Cdc42ep4\|Actr2\|Cpne8\|Rras2\|Ccdc115\|Rnd3\|Scfd1\|Rhoj\|Arhgap1\|Tubb1* |
| R-MMU-446203 | Asparagine N-linked glycosylation | 3.80 | 15/256 | *Arf1\|Copa\|F5\|Folr1\|Gfpt2\|Man2a1\|St3gal5\|Spta1\|Tuba4a\|Nus1\|Alg5\|Scfd1\|Arcn1\|Mgat2\|Tubb1* |
| WP5242 | Comprehensive IL 17A signaling | 3.80 | 9/103 | *Cdc42\|Gng12\|Il1b\|Mmp3\|Mmp9\|Orc2\|Stat3\|Rras2\|Hbb-bs* |

**Supplementary Table 3.** Top20 Significant terms detected using GO analysis with metascape in WT-PPE unique down regulated differentially expressed genes.

| **Down regulated gene** | | | | |
| --- | --- | --- | --- | --- |
| GO Term | | -log_10_(*p*-value) | InTerm_InList | Symbols |
| GO:0002250 | adaptive immune response | 16.00 | 45/506 | *B2m\|Serping1\|Ctla4\|Eomes\|Fcer2a\|H2-D1\|H2-M2\| H2-DMa\|H2-Q1\|Ighg2b\|Ighm\|Il12a\|Irf4\|Gzmm\| P2rx7\| Sh2d1a\|Stat4\|Tap1\|Tnfsf13b\|Klrk1\|Sit1\| Tnfsf13\|Clec4g\|Sla2\|Iglc2\|Ighv1-54\|Tnfrsf14\| Ighv14-3\|Ighv1-62-2\|Igkv4-55\|Ighv7-3\|Ighv2-6\| Ighv5-16\|Ighv14-2\|Ighv1-5\|Ighv1-61\|Ighv5-6\| Ighv7-1\|Ighv14-1\|Ighv3-6\|Ighv1-50\|Ighv1-55\| Ighv1-63\|Ighv2-9-1\|Ighv1-74* |
| GO:0035082 | axoneme assembly | 14.00 | 20/99 | *Foxj1\|Rsph1\|Dnaaf1\|Dnai1\|Pierce1\|Iqcg\|Spef1\|Daw1\|Odad4\|Rsph9\|Zmynd10\|Odad1\|Rsph4a\|Cfap157\|Dnai4\|Stk36\|Dnaaf6\|Dnai2\|Dnaaf3\|Dnah7c* |
| GO:0070286 | axonemal dynein complex assembly | 10.00 | 11/37 | *Dnaaf1\|Dnai1\|Daw1\|Odad4\|Zmynd10\|Odad1\|Dnai4\|Dnaaf6\|Dnai2\|Dnaaf3\|Dnah7c* |
| GO:0002706 | regulation of lymphocyte mediated immunity | 7.00 | 20/240 | *B2m\|Fcer2a\|H2-D1\|H2-M2\|H2-Q1\|Foxj1\|Ighg2b\| Il12a\| Klrc1\|Klrc2\|P2rx7\|Rasgrp1\|Sh2d1a\|Tap1\| Klrk1\|Il27ra\|Tnfsf13\|Clec4g\|Klrb1f\|Kmt5c* |
| GO:0051249 | regulation of lymphocyte activation | 6.60 | 31/548 | *B2m\|Bmp4\|Ctla4\|Efnb3\|Flt3\|H2-D1\|H2-M2\|H2-DMa\| H2-Q1\|Foxj1\|Ighm\|Il12a\|Klrc1\|Klrc2\| Nfatc2\|Rasgrp1 \|Thy1\|Tnfsf13b\|Il27ra\|Icos\|Sit1\| Twsg1\|Nrarp\|Tnfsf13\|Mzb1\|Clec4g\|Cd209a\|Tmem131l\|Tnfrsf14\|Kmt5c\|Pde5a* |
| GO:0007368 | determination of left/right symmetry | 5.60 | 13/132 | *Foxj1\|Mesp1\|Wif1\|Asb2\|Ift172\|Dnaaf1\|Dnai1\|Pierce1\|Daw1\|Odad4\|C2cd3\|Dnai2\|Dnaaf3* |
| GO:0007507 | heart development | 5.50 | 31/615 | *Axin2\|Bmp4\|Bmp7\|Cxcr4\|Cpe\|Eomes\|Eya1\|Foxj1\|Mesp1\|Myh11\|Pcnt\|Rbp4\|Dhrs3\|Sox18\|Tbx2\|Tenm4\|Sgcd\|Wif1\|Dll4\|Asb2\|Ift172\|Dnaaf1\|Dnai1\|Fuz\|Daw1\|Odad4\|Frem2\|Grhl2\|C2cd3\|Fat4\|Dnaaf3* |
| GO:0002440 | production of molecular mediator of immune response | 5.30 | 18/257 | *Ighm\|Il12a\|P2rx7\|Rasgrp1\|Tnfsf13b\|Tnfsf13\|Tnfrsf14\|Igkv8-19\|Igkv4-74\|Igkv9-124\|Igkv17-127\|Igkv4-58\|Igkv4-55\|Igkv1-99\|Igkv6-32\|Igkv4-63\|Igkv8-24\|Igkv12-46* |
| GO:0042110 | T cell activation | 4.80 | 23/418 | *B2m\|Cxcr4\|Eomes\|Bcl11a\|Flt3\|H2-D1\|H2-M2\|H2-DMa\|H2-Q1\|Il12a\|Irf4\|Myb\|P2rx7\|Rasgrp1\| Stat4\| Tnfsf13b\|Dll4\|Nrarp\|Clec4g\|Sla2\|Tnfrsf14\|Pde5a\| Cdh26* |
| GO:0002634 | regulation of germinal center formation | 4.50 | 4/10 | *H2-DMa\|Foxj1\|Tnfsf13b\|Tnfsf13* |
| GO:0010721 | negative regulation of cell development | 4.40 | 19/326 | *B2m\|Bmp4\|Bmp7\|Ctla4\|Efnb3\|Bcl11a\|Flt3\|Foxj1\|Id4\|Sema4f\|Abcc8\|Thy1\|Ulk2\|Nrarp\|Lrrc17\|Clec4g\|Tmem131l\|Syngap1\|Rgma* |
| GO:0018200 | peptidyl-glutamic acid modification | 3.80 | 5/26 | *Ggcx\|Ttll9\|Agbl4\|Agbl2\|Ttll10* |
| GO:1905331 | negative regulation of morphogenesis of an epithelium | 3.4 | 3/8 | *Bmp4\|Bmp7\|Tbx2* |
| GO:0002253 | activation of immune response | 3.1 | 18 | *Serping1\|Ctla4\|Gbp2b\|Gpr33\|H2-D1\|H2-Q1\|Ighg2b\|Ighm\|Klrc1\|Klrc2\|Blnk\|Nfatc2\|Thy1\|Klrk1\|Znfx1\|Ifi214\|Ifi213\|Ifi206* |
| GO:0140115 | export across plasma membrane | 2.9 | 6/61 | *Kcnk5\|Ralbp1\|Slc8a3\|Slc38a5\|Slc36a2\|Kcne2* |
| GO:0045165 | cell fate commitment | 2.7 | 14/283 | *Bmp4\|Eomes\|Eya1\|Irf4\|Mesp1\|Nfia\|Notch3\|Ntf3\|Sox18\|Tbx2\|Apc2\|Tenm4\|Spdef\|Dll4* |
| GO:0031295 | T cell costimulation | 2.7 | 4/27 | *Efnb3\|Tnfsf13b\|Icos\|Tnfrsf14* |
| GO:0030224 | monocyte differentiation | 2.7 | 4/27 | *Bmp4\|Ifi214\|Ifi213\|Ifi206* |
| mmu04658 | Th1 and Th2 cell differentiation - Mus musculus (house mouse) | 2.7 | 7/88 | *H2-DMa\|Il12a\|Nfatc2\|Notch3\|Stat1\|Stat4\|Dll4* |
| GO:0007420 | brain development | 2.6 | 26/706 | *Bmp4\|Bmp7\|Cxcr4\|Eomes\|Foxj1\|Id4\|Kif21b\|Notch3\|Ntf3\|Pcnt\|Pou3f3\|Ppt1\|Trp73\|Twsg1\|Ift172\|Pcdh18\|Whrn\|Odad4\|Slc4a10\|Socs7\|Duox2\|Grhl2\|Stk36\|C2cd3\|Syne2\|Fat4* |

**Supplementary Table 4.** Top20 Significant terms detected using GO analysis with metascape in *Has2*^+/−^-PPE unique up regulated differentially expressed genes.

| **Up regulated gene** | | | | |
| --- | --- | --- | --- | --- |
| GO Term | | -log_10_(*p*-value) | InTerm_InList | Symbols |
| GO:0050792 | regulation of viral process | 4.90 | 11/191 | *Csnk2b\|Ifi204\|Ltf\|Nectin2\|Axl\|Snx3\|Trim59\|Lrrc15\|Trim30c\|Isg15\|Dynlt1f* |
| GO:0036462 | TRAIL-activated apoptotic signaling pathway | 4.80 | 3/5 | *Casp8\|Fadd\|Tnfrsf10b* |
| R-MMU-5694530 | Cargo concentration in the ER | 4.50 | 5/32 | *Areg\|Cnih2\|Lman1\|Sec24a\|Mcfd2* |
| GO:0045879 | negative regulation of smoothened signaling pathway | 4.10 | 5/40 | *Runx2\|Gli2\|Enpp1\|Ptch2\|Serpine2* |
| R-MMU-73886 | Chromosome Maintenance | 4.00 | 8/126 | *Cenpc1\|Rfc2\|H2bc4\|Ctc1\|H4c8\|H4c9\|H2ac7\|H2ac12* |
| GO:0030512 | negative regulation of transforming growth factor beta receptor signaling pathway | 4.00 | 7/95 | *Adam17\|Fbn2\|Trp53\|Rasl11b\|Snx6\|Cilp\|Hdac1* |
| mmu03266 | Virion - Herpesvirus - Mus musculus (house mouse) | 3.80 | 3/10 | *Igf2r\|Nectin2\|Nectin1* |
| GO:0008340 | determination of adult lifespan | 3.60 | 5/51 | *Meg3\|Msh6\|Enpp1\|Trp53\|Cgas* |
| GO:0042476 | odontogenesis | 3.40 | 7/116 | *Runx2\|Gli2\|Enpp1\|Nectin1\|Fam20a\|Ssu2\|Hdac1* |
| GO:0035239 | tube morphogenesis | 3.30 | 20/762 | *Areg\|Casp8\|Csnk2b\|Lgr5\|Fzd1\|Gli2\|Hspg2\|Enpp1\|Aldh1a2\|Prrx2\|Smarca4\|Sox4\|Tgif1\|Fmnl3\|Zfpm2\|Plxnb2\|Myocd\|Vash1\|Arhgap22\|Itgb8* |
| WP454 | Osteoclast signaling | 3.30 | 3/14 | *Acp5\|Tnfrsf11b\|Slc9a1* |
| GO:0071824 | protein-DNA complex organization | 3.20 | 20/781 | *Cenpc1\|Haspin\|Hmgn1\|Hmg20b\|Ifi204\|Meg3\|Ptma\|Smarca4\|Trp53\|Med24\|Nap1l1\|Gnasas1\|Pih1d1\|Rian\|Zfp518b\|Dnajc9\|Myocd\|H2ac7\|H2ac12\|Hdac1* |
| R-MMU-6798695 | Neutrophil degranulation | 3.00 | 15/521 | *Csnk2b\|Cybb\|Fcna\|Folr2\|Hmox2\|Ifi204\|Igf2r\|Fabp5\|Ltf\|Rhog\|Clec12a\|Frmpd3\|Cxcl3\|Rnaset2a\|Dynlt1f* |
| GO:0010470 | regulation of gastrulation | 3.00 | 3/18 | *Hnf4a\|Tgif1\|Phldb2* |
| GO:1902105 | regulation of leukocyte differentiation | 3.00 | 12/367 | *Casp8\|Fadd\|Fanca\|Hmgb3\|Tnfrsf11b\|Smarca4\|Sox12\|Sox4\|Tnfsf4\|Axl\|Tmem176b\|Malt1* |
| GO:0060348 | bone development | 3.00 | 9/223 | *Acp5\|Runx2\|Hspg2\|Ltf\|Enpp1\|Trp53\|Ctc1\|Slc38a10\|Adamts7* |
| GO:0010659 | cardiac muscle cell apoptotic process | 2.90 | 3/19 | *Trp53\|Myocd\|Dynlt1f* |
| R-MMU-201681 | TCF dependent signaling in response to WNT | 2.80 | 7/150 | *Csnk2b\|Lgr5\|Fzd1\|Smarca4\|Sox4\|Psmd10\|Hdac1* |
| R-MMU-72165 | mRNA Splicing - Minor Pathway | 2.80 | 4/45 | *Snrnp40\|Polr2d\|Snrpf\|Sf3b4* |
| GO:0009615 | response to virus | 2.80 | 11/338 | *Fadd\|Gli2\|Ifi204\|Acod1\|Tnfsf4\|Ddx41\|Ifi27l2a\|Cgas\|Itgb8\|Trim30c\|Isg15* |

**Supplementary Table 5.** Top20 Significant terms detected using GO analysis with metascape in *Has2*^+/−^-PPE unique down regulated differentially expressed genes.

| **Down regulated gene** | | | | | |
| --- | --- | --- | --- | --- | --- |
| GO Term | | -log_10_(*p*-value) | InTerm_InList | Symbols |  |
| GO:0031669 | cellular response to nutrient levels | 4.10 | 13/226 | *Glul\|Kat2b\|Pck1\|Usf1\|Wdr45\|Tsc1\|Atg4b\|Map1lc3b\|Gabarapl2\|Flcn\|Zfyve1\|Prkag3\|Scd4* |  |
| GO:0043393 | regulation of protein binding | 4.00 | 12/201 | *Bdnf\|Bmp2\|Cldn5\|Aktip\|Mfng\|Xirp1\|Prkn\|Dnajb2\|Errfi1\|Ip6k2\|Cblb\|Efhb* |  |
| R-MMU-1632852 | Macroautophagy | 3.80 | 9/120 | *Prkn\|Wdr45\|Tsc1\|Chmp4c\|Atg4b\|Map1lc3b\|Pink1\|Gabarapl2\|Prkag3* |  |
| GO:0048738 | cardiac muscle tissue development | 3.70 | 12/216 | *Bmp2\|S1pr1\|Jarid2\|Myh6\|Ryr2\|Ttn\|Xirp1\|Bves\|Tsc1\|Akap13\|Arid2\|Alpk3* |  |
| mmu04152 | AMPK signaling pathway - Mus musculus (house mouse) | 3.70 | 9/127 | *Igf1r\|Irs3\|Pck1\|Ppargc1a\|Foxo3\|Tsc1\|Pfkfb3\|Prkag3\|Scd4* |  |
| GO:0043269 | regulation of monoatomic ion transport | 3.50 | 20/516 | *Casq2\|Slc6a4\|Kcnj15\|Kcnj5\|Fxyd3\|Prkca\|Plcg1\|Ptpn22\|Ryr2\|Stim1\|Slc30a1\|Tesc\|Shank3\|Sln\|Saraf\|Hbp1\|Mchr1\|Efhb\|Lrrc26\|Gpd1l* |  |
| GO:0090258 | negative regulation of mitochondrial fission | 3.50 | 3/9 | *Ppargc1a\|Prkn\|Pink1* |  |
| GO:0008285 | negative regulation of cell population proliferation | 3.50 | 26/772 | *Bdnf\|Bmp2\|Bmpr2\|Cd37\|Gata2\|Slc6a4\|Jarid2\|Ppp1r15a\|Kat2b\|Prkca\|Ppargc1a\|Xdh\|Xirp1\|Tspan32\|Dnajb2\|Tesc\|Tsc1\|Arid2\|Ripor2\|Cblb\|Plk5\|Appl2\|Flcn\|Zbtb16\|Acvr1c\|Intu* |  |
| GO:0044242 | cellular lipid catabolic process | 3.40 | 11/197 | *Prdx6\|Auh\|Pck1\|Plcg1\|Smpd2\|Gdpd1\|Pnpla2\|Nudt7\|Gpihbp1\|Gba2\|Acad12* |  |
| GO:0043462 | regulation of ATP-dependent activity | 3.30 | 6/63 | *Myh6\|Ryr2\|Dnajb2\|Tsc1\|Sln\|Pot1b* |  |
| GO:0050860 | negative regulation of T cell receptor signaling pathway | 3.20 | 4/25 | *Elf1\|Ptpn22\|Dusp3\|Cblb* |  |
| GO:0060426 | lung vasculature development | 3.10 | 3/12 | *Bmp2\|Bmpr2\|Errfi1* |  |
| GO:0031400 | negative regulation of protein modification process | 2.9 | 17/454 | *Bmp2\|Ppp1r15a\|Kat2b\|Pecam1\|Prkca\|Ppargc1a\|Ptpn22\|Ube2b\|Xdh\|Prkn\|Dnajb2\|Dusp3\|Errfi1\|Cmya5\|Cblb\|Flcn\|Gpd1l* |  |
| GO:0032868 | response to insulin | 2.8 | 10/198 | *Igf1r\|Irs3\|Kat2b\|Pck1\|Prkci\|Usf1\|Tsc1\|Errfi1\|Trarg1\|Acvr1c* |  |
| R-MMU-9840310 | Glycosphingolipid catabolism | 2.8 | 4/31 | *Smpd2\|Glb1l3\|Gba2\|Glb1l2* |  |
| GO:0060419 | heart growth | 2.8 | 5/53 | *S1pr1\|Jarid2\|Ttn\|Akap13\|Arid2* |  |
| R-MMU-8854214 | TBC/RABGAPs | 2.8 | 4/32 | *Tsc1\|Map1lc3b\|Gabarapl2\|Tbc1d16* |  |
| GO:0051960 | regulation of nervous system development | 2.8 | 20/596 | *Bdnf\|Bmp2\|Bmpr2\|Gata2\|Nkx6-2\|Myo5b\|Oprm1\| Prkca\|Prkci\|Sema4g\|Shank3\|Parp6\|Casz1\|Fbxo31\|Jade2\|Trim11\|Appl2\|Lrtm1\|Lingo4\|Tnik* |  |
| mmu01521 | EGFR tyrosine kinase inhibitor resistance - Mus musculus (house mouse) | 2.8 | 6/79 | *Araf\|Igf1r\|Il6ra\|Prkca\|Plcg1\|Foxo3* |  |
| mmu04928 | Parathyroid hormone synthesis, secretion and action - Mus musculus (house mouse) | 2.7 | 7/108 | *Araf\|Jund\|Lrp5\|Prkca\|Akap13\|Pde4c\|Adcy5* |  |
